# Network analytics for drug repurposing in COVID-19

**DOI:** 10.1093/bib/bbab490

**Published:** 2021-12-02

**Authors:** Nicoleta Siminea, Victor Popescu, Jose Angel Sanchez Martin, Daniela Florea, Georgiana Gavril, Ana-Maria Gheorghe, Corina Iţcuş, Krishna Kanhaiya, Octavian Pacioglu, Laura lona Popa, Romica Trandafir, Maria Iris Tusa, Manuela Sidoroff, Mihaela Păun, Eugen Czeizler, Andrei Păun, Ion Petre

**Affiliations:** Faculty of Mathematics and Computer Science, ICUB, University of Bucharest, Romania and Department of Bioinformatics, National Institute of Research and Development for Biological Sciences, Romania; Department of Information Technologies, Åbo Akademi University, Turku, Finland; Departamento de Sistemas Informáticos, Universidad Politécnica de Madrid, Spain; Department of Bioinformatics, National Institute of Research and Development for Biological Sciences, Romania; Department of Bioinformatics, National Institute of Research and Development for Biological Sciences, Romania; Department of Bioinformatics, National Institute of Research and Development for Biological Sciences, Romania; Department of Arctic and Antarctic Research, National Institute of Research and Development for Biological Sciences, Romania and Romanian Young Academy, University of Bucharest, Romania; Department of Information Technologies, Åbo Akademi University, Turku, Finland; Department of Bioinformatics, National Institute of Research and Development for Biological Sciences, Romania; Department of Microbiology and Immunology, Faculty of Biology, University of Bucharest, Bucharest, Romania and Department of Bioinformatics, National Institute of Research and Development for Biological Sciences, Bucharest, Romania; Department of Bioinformatics, National Institute of Research and Development for Biological Sciences, Bucharest, Romania, and Department of Mathematics and Computer Science, Technical University of Civil Engineering Bucharest, Romania; Department of Arctic and Antarctic Research, National Institute of Research and Development for Biological Sciences, Romania; Department of Arctic and Antarctic Research, National Institute of Research and Development for Biological Sciences, Romania; Department of Bioinformatics, National Institute of Research and Development for Biological Sciences, Romania, and Faculty of Administration and Business, Research Institute of the University of Bucharest–ICUB, University of Bucharest, Romania; Department of Bioinformatics, National Institute of Research and Development for Biological Sciences, Romania; Faculty of Mathematics and Computer Science, ICUB, University of Bucharest, Romania, and Department of Bioinformatics, National Institute of Research and Development for Biological Sciences, Romania; Department of Mathematics and Statistics, University of Turku, 20014 Turku, Finland and National Institute of Research and Development for Biological Sciences, 296 Independenţei Bd. District 6, 060031 Bucharest, Romania

**Keywords:** COVID-19, drug repurposing, network biology, network controllability, host factors

## Abstract

To better understand the potential of drug repurposing in COVID-19, we analyzed control strategies over essential host factors for SARS-CoV-2 infection. We constructed comprehensive directed protein–protein interaction (PPI) networks integrating the top-ranked host factors, the drug target proteins and directed PPI data. We analyzed the networks to identify drug targets and combinations thereof that offer efficient control over the host factors. We validated our findings against clinical studies data and bioinformatics studies. Our method offers a new insight into the molecular details of the disease and into potentially new therapy targets for it. Our approach for drug repurposing is significant beyond COVID-19 and may be applied also to other diseases.

## 1 Introduction

**Figure 1:**
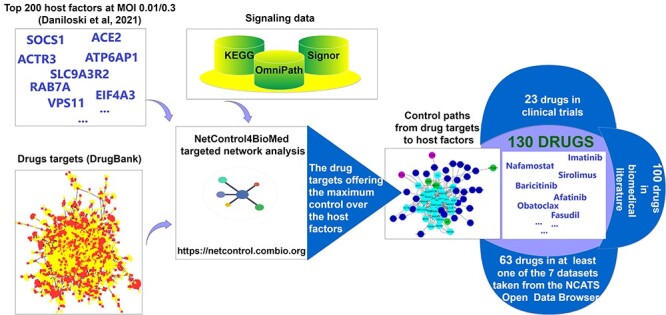
Study design of network controllability for drug repurposing. We included all the approved and investigational small molecule drugs, except for those illicit or nutraceutical. For each drug (red in the bottom-left network) we identified their drug targets [8] (yellow in the bottom-left network). We also included the top 200 host factors required for SARS-CoV-2 infection found by [4] for viral loads at MOI 0.01 and 0.3, and all protein–protein-directed interactions from KEGG [13], OmniPath [14] and SIGNOR [15]. Using the *NetControl4BioMed* platform [16], we identified all control paths of length at most 3 from drug targets to host factors. We ranked the drugs based on the number of host factors they can control through any of their targets.

The COVID-19 pandemic has caused more than 234 million infections worldwide, with more than 4.8 million deaths (as of October 2021) [1]. Several vaccines are available since the fall 2020 and this has helped curtail the infection rate worldwide. Many drugs are investigated in clinical trials, and several have been approved or recommended for use, including remdesivir, dexamethasone and some combinations of monoclonal antibodies. It remains of major interest to gain a system-level understanding of the molecular details of the disease and to translate them into effective treatment strategies for the disease. Such data are increasingly available, for example on the human proteins that associate with SARS-CoV-2 proteins upon infection [2], on potential drug targets based on mechanisms preserved in SARS-CoV-1, SARS-CoV-2 and MERS [3], and on the host genes critical for the infection with SARS-CoV-2 [4]. Computational network-based methods can integrate SARS-CoV-2 data into comprehensive models, as described in [5] through a combination of network diffusion/proximity and artificial intelligence. Also, [6] has integrated data from multiple sources to provide a network-based systemic understanding of protein–protein interactions (PPI), virus–host interactions, biological processes, drugs and symptoms related to COVID-19. Additional viruses were included in [7] to demonstrate the systemic nature of SARS-CoV-2. The recent results of [4] offer a dataset with a strong therapeutic potential. Through a number of genome-scale loss-of-function screens, it identified and ranked host factors required for the SARS-CoV-2 viral infection, i.e. genes whose loss of function confers resistance to the infection. It identified the relevant host factors both at a lower viral load of multiplicity of infections (MOI) 0.01 and a higher one of MOI 0.3. Out of the 200 top ranked genes, only a small fraction are drug targetable [8] (24 from the MOI 0.01 host factors and 23 from the MOI 0.3 ones). We asked whether more of the host factors can be targeted through drugs acting upstream of them. To investigate this question, we built two directed PPI networks (one for each MOI dataset) integrating interaction data upstream of the essential SARS-CoV-2 host factors (separately for MOI 0.01 and MOI 0.3) and interaction data downstream of all currently available drug targets from DrugBank [8]. We analyzed the networks in the framework of control theory [9] and sought to identify minimal combinations of drug targets that offer control (through cascading signals in the interaction network) over the essential host factors (Figure 1). Network controllability has been demonstrated successfully in several studies: to identify repurposable drugs for a form of leukemia [10], to identify the contribution of individual neurons in the locomotion of *Caenorhabditis elegans* [11], and for personalized drug repurposing in breast cancer and COVID-19 [12]. We focused on short control paths from drug targets to host factors to minimize the possible dissipation of the drug’s influence along the path. Control here is understood in the sense of structural network controllability and its results offer a systemic view on how to influence the host factors simultaneously through available drug targets. Moreover, the resulting drug targets (and the drugs acting on them) can be ranked with respect to how many host factors each can control in the network, independently of the other drug targets. Combining these results offers a number of drugs and drug combinations that are potentially efficient at influencing the host factors. The results of structural controllability are qualitative: they offer therapeutically promising drugs and drug combinations, but do not offer numerical indications on the optimal concentrations and possible toxicities. We compared our findings with clinical trials data and bioinformatics analyses. We found a number of drugs that have been investigated in clinical trials, but also some new ones, not yet studied in connection to COVID-19.

Our key advance, therefore, is a new system-level insight into the molecular details of the disease that is able to offer a significant number of potentially efficient therapies based on drug repurposing.

## 2 Results

### 2.1 COVID-19–specific directed PPI networks

We constructed directed PPI networks around the host factors identified in [4] to be required for SARS-CoV-2 infection. We constructed two different interaction networks, one for each set of top-ranked host factors reported by [4], one obtained at a low (0.01) MOI, the other at a high (0.3) MOI. From each experiment we included the top 200 ranked genes, whose loss-of-function mutations led to enrichment in their pools. We also included all drug targets from DrugBank [8]. For the interaction data we used KEGG [13], OmniPath [14] and SIGNOR [15]. We only included the directed PPIs found in these databases. The networks were well connected (a single connected component in one, two in the other), compact (diameter equal to 10) and rich in interactions (over 20 000 interactions). Each network included more than 1000 drug targets and about one-third of the top 200 host factors (70 for MOI 0.01 and 62 for MOI 0.3; the others were further from the drug targets in our networks). A description of how the networks were generated and their topological analysis is available in Supplementary Information, Section 1.

### 2.2 Network-based identification of repurposable drug targets

We performed structural target controllability analysis on the two interaction networks, considering as control targets the MOI-specific top 200 host factors of [4]. The mathematics of structural target controllability is discussed briefly in Supplementary Information, Section 2. We used as preferred control inputs the drug-targetable genes from DrugBank [8]. In each analysis, we identified the drug(s) whose targets control the most host factors. The analysis is based on a stochastic search for paths from drug targets to host factors and so, repeated analyses identified multiple results on the same network. For each network, we repeated the analysis until three consecutive runs identified no new drugs. The analyses were run on the *NetControl4BioMed* platform [16]. The goal was to find directed paths from the drug targets to the set of host factors that are structural target control paths in the sense of control theory. To achieve this, a Greedy algorithm is applied to solve a directed graph matching problem using a minimum number of input nodes, selected as much as possible from among the set of drug targets. Details on the algorithms are in [17].

**Figure 2:**
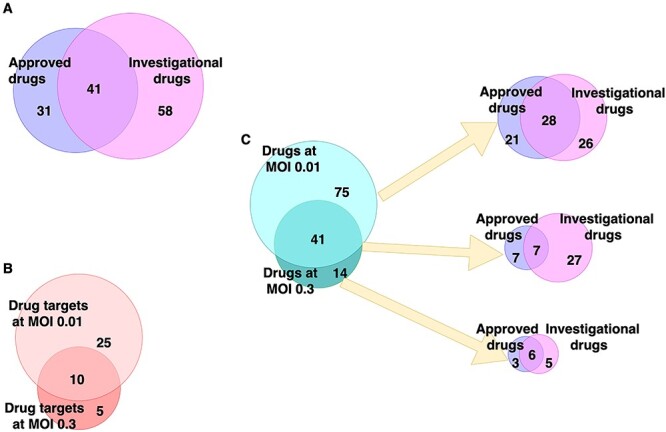
Drug categories. (**A**) The overlap between all approved or investigational drugs. (**B**) The overlap between drug targets in both networks. (**C**) The overlap between approved and investigational drugs for each network and their intersection.

We found that the host factors can be controlled though 35 drug targets for the MOI 0.01 network and through 15 for the MOI 0.3 network (see Figure 1 in Supplementary Information), of which 10 are common for both networks: ACTB, AKT1, ATM, ATP6AP1, CSNK2A1, CDK2, EGF, MAPK14, MTOR and TP53. Of these 10, two are essential host factors for the SARS-CoV-2 infection [4]: ACTB (in the MOI 0.3 experiment) and ATP6AP1 (in both MOI experiments). Additionally, ATP6AP1 is also known to interact directly with SARS-CoV-2 [2]. We identified 116 drugs from the analysis of the MOI 0.01 network acting on these targets and 55 drugs from the analysis of the MOI 0.3 network, with 41 common to both of them (Figure 2).

**Figure 3:**
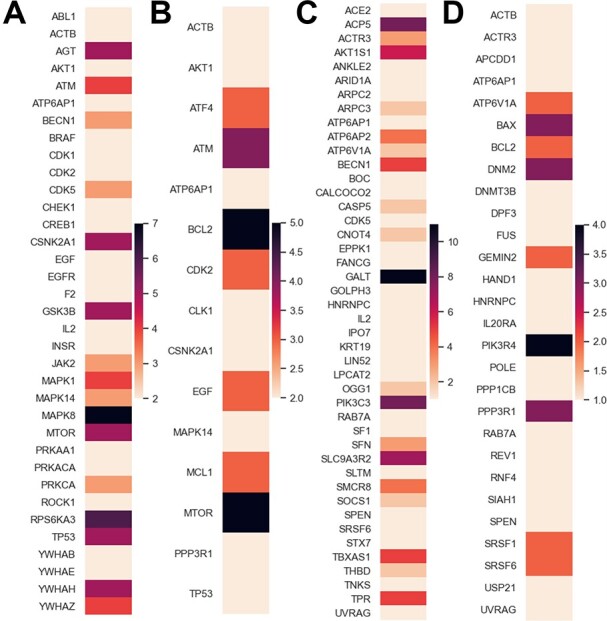
drug targets and the number of host factors they can control. (**A**) MOI 0.01, (**B**) MOI 0.3. Host factors for the SARS-CoV-2 infection and the number of drug–target genes that can be used to control them. (**C**) MOI 0.01, (**D**) MOI 0.3.

Not all host factors could be controlled by repurposed drugs: only 44 MOI 0.01 host factors and only 28 MOI 0.3 host factors were included in the control results found by our analysis (see Figure 1 in Supplementary Information). In the MOI 0.01 analysis, the gene that turned out to be the easiest to control is GALT: it can be controlled by more than 10 drug targets (Figure 3). On the other hand, the well-known ACE2 is one of the hardest to target: it can be controlled by only one drug target, namely AGT.

A ranking of the theoretical efficacy of a drug target can be done on the basis of how many host factors were found to control in our analyses (Figure 3**A** and **B**). Also, a ranking of how easily controllable a host factor is can be done on the basis of how many drug targets can control it (Figure 3**C** and **D**).

For the MOI 0.01 host factors, the top-ranked drug target was MAPK8, which controls seven host factors. Interestingly, MAPK8 was not identified as a control input in our analyses for the MOI 0.3 list of host factors. For MAPK8 we found two drugs acting on it: minocycline and tamoxifen. The top-ranked drug targets occurring in both sets of results is MTOR, controlling five host factors from each list, and ATM, controlling four host factors from each list. There are several drugs that act on MTOR: everolimus, pimecrolimus, ridaforolimus, rimiducid, SF1126, sirolimus, temsirolimus and XL765.

For the 0.3 MOI host factors, the top-ranked drug target, in addition to MTOR, is BCL2, which regulates cell death and attenuates inflammation [18]. The following drugs act on this target: apoptone, dexibuprofen, docetaxel, eribulin, ibuprofen, isosorbide, navitoclax, obatoclax, paclitaxel, paclitaxel docosahexaenoic acid, rasagiline and venetoclax.

### 2.3 Control paths

Our analyses identified the structural target control paths from the drug targets to the host factors. We obtained 108 control paths for the MOI 0.01 network and 43 control paths for the MOI 0.3 network, 8 of them being common to both networks. We ran an enrichment analysis with PANTHER and GO biological process complete [19–21] with the false discovery rate $P < 0.05$. To avoid spurious results, we only included the control paths with three or more proteins. The most specific processes in the enrichment hierarchy that include all proteins involved in each control path are presented in Tables 1 and 2. The results show many of the enriched pathways to be part of the cellular response to external stimuli and to stress, as well as of the regulation of cell death.

**Table 1:** Enrichment analysis results for control paths with more than two proteins for the MOI 0.01 networks

**Control path**	**GO biological process complete**
TP53 $\rightarrow $ CASP1 $\rightarrow $ CASP5	apoptotic signaling pathway, cellular response to external stimulus, cellular response to environmental stimulus
MAPK1 $\rightarrow $ CASP8 $\rightarrow $ CASP1 $\rightarrow $ CASP5	cell death, cellular response to external stimulus, apoptotic process, regulation of response to external stimulus, regulation of response to stress
ROCK1 $\rightarrow $ BECN1 $\rightarrow $ PIK3C3	cell division
AGT $\rightarrow $ BECN1 $\rightarrow $ PIK3C3	cellular response to external stimulus
TP53 $\rightarrow $ MCL1 $\rightarrow $ BECN1 $\rightarrow $ PIK3C3	cellular response to stress
AGT $\rightarrow $ REN $\rightarrow $ ATP6AP2	circulatory system process, endocrine process
GSK3B $\rightarrow $ BCL2 $\rightarrow $ BECN1	mitochondrion organization, regulation of autophagy, regulation of apoptotic signaling pathway
GSK3B $\rightarrow $ TBK1 $\rightarrow $ CALCOCO2	positive regulation of cellular catabolic process, regulation of autophagy
ABL1 $\rightarrow $ WAS $\rightarrow $ ARPC3	Regulation of protein-containing complex assembly, positive regulation of cellular component biogenesis, positive regulation of organelle organization, regulation of cytoskeleton organization, regulation of actin filament-based process, regulation of anatomical structure size, regulation of actin filament organization, supramolecular fiber organization, actin filament-based process
PRKACA $\rightarrow $ CREB1 $\rightarrow $ ACP5	response to cytokine
EGFR $\rightarrow $ BECN1 $\rightarrow $ PIK3C3	response to extracellular stimulus, cellular response to external stimulus, endocytosis, regulation of cell cycle process
MAPK8 $\rightarrow $ BECN1 $\rightarrow $ PIK3C3	response to extracellular stimulus, cellular response to external stimulus, response to starvation

**Table 2:** Enrichment analysis results for the control paths with more than two proteins for the MOI 0.3 network

**Control paths**	**GO biological process complete**
ATF4 $\rightarrow $ BCL2 $\rightarrow $ BAX	Cell death, response to endoplasmic reticulum stress, intrinsic apoptotic signaling pathway, response to abiotic stimulus, response to light stimulus, response to UV, immune system development, sensory organ development, animal organ morphogenesis, negative regulation of cell death, negative regulation of neuron death, regulation of response to stress
MCL1 $\rightarrow $ BCL2 $\rightarrow $ BAX	Cell death, signal transduction in absence of ligand, negative regulation of cell death, cellular homeostasis, intrinsic apoptotic signaling pathway, negative regulation of cell communication, negative regulation of signaling, regulation of apoptotic signaling pathway, cellular response to DNA damage stimulus, regulation of intrinsic apoptotic signaling pathway, regulation of neuron death, regulation of catabolic process
AKT1 $\rightarrow $ BECN1 $\rightarrow $ PIK3R4	cellular response to external stimulus
MCL1 $\rightarrow $ BCL2 $\rightarrow $ BECN1 $\rightarrow $ PIK3R4	cellular response to stress
CDK2 $\rightarrow $ BRCA2 $\rightarrow $ POLH $\rightarrow $ REV1	cellular response to stress, DNA metabolic process, macromolecule biosynthetic process, DNA replication
CDK2 $\rightarrow $ PCNA $\rightarrow $ POLH $\rightarrow $ REV1	cellular response to stress, DNA metabolic process, macromolecule biosynthetic process, DNA replication
MAPK14 $\rightarrow $ CSNK2A2 $\rightarrow $ HNRNPC	negative regulation of catabolic process
TP53 $\rightarrow $ DKK1 $\rightarrow $ WNT3A $\rightarrow $ APCDD1	negative regulation of response to stimulus
BCL2 $\rightarrow $ CASP3 $\rightarrow $ PPP3R1 $\rightarrow $ DNM2	neurogenesis
CLK1 $\rightarrow $ SRPK1 $\rightarrow $ SRSF6	regulation of RNA splicing
ATF4 $\rightarrow $ BCL2 $\rightarrow $ BECN1 $\rightarrow $ PIK3R4	response to extracellular stimulus, cellular response to external stimulus, response to starvation, cellular response to stress
BCL2 $\rightarrow $ BECN1 $\rightarrow $ PIK3R4	response to extracellular stimulus, cellular response to external stimulus, response to starvation, regulation of cell cycle process
BCL2 $\rightarrow $ CASP3 $\rightarrow $ PPP3R1	tissue morphogenesis, tube morphogenesis

### 2.4 Potentially repurposable drugs

Using data from DrugBank [8] we found 130 drugs acting on the drug targets resulting from the two MOI datasets and their corresponding control analyses, including 41 that act on the 10 drug targets common to the two analyses. The results are listed in Supplementary Table 1. Of these drugs, 72 are approved for at least one condition according to [8]. Most are drugs used in oncology, and we also found direct inhibitors of thrombin, anti-inflammatory, estrogens and mood-stabilizers. In terms of their cellular location of action, most drug targets are located in the surrounding of cytoplasmic vesicles, and in the cell–substrate junction between the cell and extracellular matrix (see Figure 2 in Supplementary Information).

#### Antineoplastic and immunomodulating agents

Most drugs identified in our analyses are antineoplastic agents and, within this group, most of them are protein kinase inhibitors. Some of the drugs we identified are not yet approved, and therefore do not have ATC codes (e.g. alvocidib, gensitein, pelitinib, seliciclib). The approved drugs are summarized in Table 3, grouped by their targets in our networks. Some of these drugs were included in this list through some of their secondary targets (e.g. brigatinib on INSR and dasatinib on MAPK14), and other drugs are included through several of their targets, potentially indicating increased efficacy (e.g. bosutinib and brigatinib).

**Table 3:** The top-ranked antineoplastic and immunomodulating agents identified in our analyses and their targets

**Control inputs**	**Drugs targeting the control inputs**
ABL1	Bosutinib, brigatinib, dasatinib, imatinib, nilotinib, ponatinib, regorafenib
BRAF	Dabrafenib, encorafenib, regorafenib, ripretinib, sorafenib, vemurafenib
CDK2	Bosutinib
EGFR	Afatinib, brigatinib, dacomitinib, erlotinib, gefitinib, icotinib, lapatinib, neratinib, olmutinib, osimertinib, vandetanib, zanubrutinib
INSR	Brigatinib
JAK2	Entrectinib, fedratinib, ruxolitinib, zanubrutinib
MAPK14	Dasatinib
MTOR	Everolimus, ridaforolimus, temsirolimus
PRKCA	Midostaurin

Some of these inhibitors have been investigated in connection with several viruses, including SARS-CoV-2. The EGFR inhibitors, which are principally used in non-small cell lung cancers or breast cancers, may act on SARS-CoV-2 virus replication [22], whereas JAK2 inhibitors could act on SARS-CoV-2 cytokine storm because IL-6 and GM-CSF, which are stimulated in this infection, depend on JAK2 signaling [23]. The mTOR pathway can be targeted by many viruses (e.g. IAV, MERS) to promote their replication. Its inhibition was shown to lead to a decrease in SARS-CoV-2 virus production [24].

Other antineoplastic agents found in our analyses, docetaxel, paclitaxel, eribulin, venetoclax, are not protein kinase inhibitors. They were identified in the network corresponding to the MOI 0.3 experiment.

We also obtained selective immunosuppressants such as: baricitinib, sirolimus, tofacitinib and a calcineurin inhibitor, voclosporin. Sirolimus, a drug used to prevent organ rejection in renal transplants and suggested for COVID-19 also in [25], was identified in both network analyses, due to its inhibitory effect on MTOR, whereas baricitinib and tofacitinib were identified only in the MOI 0.01 network analysis, due to their inhibitory action on JAK2. Both are used in rheumatoid arthritis, and baricitinib has Food and Drug Administration (FDA) approval for use in COVID-19. We obtained voclosporin only for the MOI 0.3 network because of its inhibitory effect on PPP3R1 subunit of calcineurin, which leads to blocking the transcription of early inflammatory cytokines [8].

#### Antithrombotic agents

There is a known link between COVID-19 and coagulopathy: in many severe cases, disseminated intravascular coagulation is observed [26], which is associated with higher mortality [27]. Our analysis revealed several compounds in use as antithrombotic agents (argatroban, bivalirudin, dabigatran etexilate and ximelagatran), all based on their inhibitory effect on prothrombin. We also obtained investigational drugs (flovagatran, gabexate, nafamostat) and an F2 agonist, in other words an antihemorrhagic (kappadione). Kappadione is a vitamin K derivative, and vitamin K has an important role in activating both pro- and anti-clotting factors in the liver, and extra-hepatic vitamin K deficiency has been observed in COVID-19 patients [28].

One drug present on our list that cannot be used in patients with COVID-19 is proflavine because it has only topical use as a disinfectant, and it is toxic and carcinogenic in mammals [8]. It ends up being included in our results through its targeted action on F2, which was found to control several host factors.

#### Estrogens

Estrogens being included in our list is consistent with COVID-19 mortality being not only higher in the elderly, but also in men compared with women [29]. One cause of these differences may be estradiol, several of which are included in our results. Estradiol regulates several pathways in the immune system [30].

Our analysis revealed BECN1 as being relevant, a gene that plays a role in autophagy and may also play a role in antiviral host defense [8]. Using these drugs carries the risk of thromboembolism, even if they may increase the expression/activity of ACE2 in the adipose tissue and in the kidney [31]. Some studies recommend estradiol for further investigation: 68 466 cases were analyzed in [32] with the results indicating that estradiol decreased COVID-19 fatality. However, estradiol has multiple functions in the body, so its potential adoption in COVID-19 should be cautiously verified further.

Other compounds identified by our analyses are discussed in Supplementary Information, Section 3.

### 2.5 Drug combinations

We used the results of the target controllability analyses to identify potential drug combinations (in Supplementary Table 2). For each analysis, we identified the combinations of two and three drugs whose drug targets control together the highest number of host factors. We only considered the combinations of drugs whose sets of drug targets don’t fully overlap (i.e. each drug has at least one specific drug target not shared with the other drugs).

In the case of the MOI 0.01 network, we found 23 unique combinations of two drugs with a maximum number of controlled host factors. Some of them included aspirin and one of the drugs acting on MTOR.

Other drug combinations are centered on alvocidib, a drug investigated for use in non-small lung cancer. Its combinations are with enzastaurin, minocycline, perifosine, tamoxifen, phenethyl isothiocyanate and quercetin. Enzastaurin and perifosine are AKT1 inhibitors and may be associated with CDK4/6 inhibitors (two of the alvocidib targets) [33].

In the case of the MOI 0.3 network, we found 42 combinations of two drugs with a maximum number of controlled host factors. Dasatinib and ellagic acid are used in multidrug-resistant tumors [34], whereas dasatinib plus quercetin has been shown to be useful in relieving intestinal senescence and inflammation [35]. Other combinations obtained are those with bosutinib and any of the drugs that act on BCL2.

Our analysis also gave BCL2 inhibitors associated with SF1126 or sirolimus (both MTOR inhibitors), and this combination of BCL2 with MTOR inhibitors is used in resistant acute lymphoblastic leukemia [36]. The other drug combinations include tesevatinib with MTOR inhibitors or caffeine.

### 2.6 Experimental and clinical validation

#### 2.6.1 Validation using the NCATS COVID-19 OpenData Portal

Our first approach to validate the results was to search for them on the OpenData Portal [37]. This data platform collects validation data on potential COVID-19 drugs in terms of viral entry, viral replication, *in vitro* infectivity, life virus infectivity and human cell toxicity. The platform lists both complete and incomplete results, e.g. untested drugs, or drugs with parts of the test results not yet available. We only focused on the results on SARS-CoV-2 and excluded the analyzes made on MERS and SARS. We also excluded human cell toxicology tests because we considered these types of tests more as a selection measure between two drugs with similar effects and not as a validation of activity because a drug can be inactive and can be very toxic or, conversely, can be well tolerated. So, we included the Spike-ACE2 PPI assay and counter assay, ACE2, TMPRSS2 and 3CLpro enzymatic activity tests and SARS-CoV-2 cytophatic effect and counter assay.

**Figure 4:**
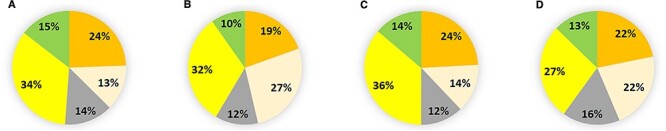
Drug classification based on their activity according to NCATS COVID-19 OpenData Portal [37]. (**A**) Drugs identified in either of the two MOI networks. (**B**) Drugs identified in both MOI networks. (**C**) Drugs identified in the MOI 0.01 network. (**D**) Drugs identified in the MOI 0.3 network. **Color code**: green – active, orange – not tested, cream – inactive, but not tested in all assays, gray – inactive, yellow – active in the counter assay.

We divided our results into the five categories used in the portal. The first category (in green in Supplementary Table 1) is that of active drugs in at least one of the following five tests: Spike-ACE2 PPI, SARS-CoV-2 cytophatic effect and enzymatic activity of ACE2, TMPRSS2 and 3CLpro. We also set the condition that, if it is active in a test, it should be inactive in the corresponding counter-test. The second category (in yellow in Supplementary Table 1) contains active drugs in a counter assay, regardless of its status in the corresponding assay. The third category (in gray in Supplementary Table 1) contains drugs that are inactive in all tests. The fourth (in orange in Supplementary Table 1) includes drugs that haven’t been tested at all. Finally, the fifth category (in cream in Supplementary Table 1) contains partially tested drugs that have been found inactive in the tests performed. We illustrated the distribution of our results among these categories in Figure 4. The largest proportion is that of the drugs active in counter assays.

We found the following results in the “active drug” category: afatinib, baricitinib, docetaxel, entrectinib, enzastaurin, erlotinib, fasudil, gabexate, imatinib, minocycline, nafamostat, navitoclax, ponatinib, proflavine, R-1487, ruxolitinib, sirolimus, suramin and venetoclax.

We also found several drugs active both in the assay, as well as in the counter assay for the Spike-ACE2 PPI, and several drugs in the counter assay for cytopathic effect (see Figure 3 in Supplementary Information).

We also investigated the results on the enzymatic activity of ACE2, TMPRSS2 and 3CLpro (see Figure 4 in Supplementary Information) and found two drugs that act on ACE2 and TMPRSS2, in other words on viral entry, namely ruxolitinib and fasudil. Ruxolitinib was obtained in our study on the MOI 0.01 network as a JAK2 inhibitor. It has been previously recommended for investigation in COVID-19 [38] and it has been included in clinical trials with little success [39].

Fasudil was also obtained in the MOI 0.01 network, targeting ROCK1 and PRKACA. It may help in the associated acute lung injury and acute respiratory distress syndrome, and, in addition to its protective effect against lung damage, it possesses antifibrotic properties and the ability to upregulate ACE2 [40]. We also found (in the MOI 0.3 network) a drug that acts on the enzymatic activity of ACE2 and 3CLpro: venetoclax, a BCL2 inhibitor.

#### 2.6.2 In vitro validation

About a quarter of the drugs we identified were already reported in the experimental literature. One of them is nafamostat, which inhibited mediated entry into host cells with an efficiency approximately 15 times higher than camostat mesylate [41] and also blocked infection of Calu-3 cells with an effective concentration (EC) 50 around 10 nM, while a significantly higher dose (EC50 around 30 $\mu $M) was required for VeroE6/TMPRSS2 [42].

Other drugs were tested on several cell types and were found to be active. For example, nilotinib inhibits SARS-CoV-2 in Vero-E6 cells and Calu-3 cells [43].

Another drug is obatoclax, which can inhibit SARS-Cov-2 replication in vitro, in human nasal epithelial cells [44], and in Vero-E6 cells [45]. Tamoxifen not only showed a 100-fold reduction in viral load in human cells *in vitro* [4], but also showed inhibitory activity in Vero E6 cells [46].

There are also drugs with conflicting results based on the type of cells used, such as bosutinib, which is active in Huh 7, active in Vero E6, but not as potent, and inactive in Cacao-2 E6 and in iAEC2 [47].

BRAF inhibitors (dabrafenib, regorafenib and sorafenib) as well as baricitinib lead to increased virus growth [48]. Regorafenib may play a role in the receptor-mediated host response to SARS-CoV-2 [49], dabrafenib may inhibit SARS-CoV-2 infection [50] and sorafenib could prevent *in vitro* replication [22]. According to [49], sorafenib has antiviral activities, but the cytotoxic and antiviral IC50 values are close. Baricitinib prevented progression to a severe form by modulating the patient’s immune landscape in a 20-case study [51] and its administration resulted in an improvement in respiratory function in a study of 62 patients who received baricitinib and coticosteroids compared with 50 patients who received only corticosteroids [52]. In another study [53] in rhesus macaques, baricitinib suppressed the production of proinflammatory cytokines, maintained innate antiviral responses and SARS-CoV-2 T cells and limited the recruitment of neutrophils to the lungs and neutrophil cell death (NETosis). 

**Figure 5:**
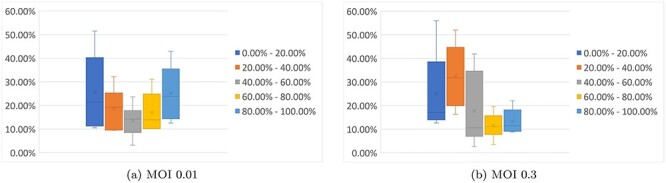
The robustness analysis results. The box plots represent how often various drugs were identified in the control analyses we ran, with the search done in various parameter settings: the host factors selected to be the top 50/100/150/200/250/300 ranked in the [4] datasets; the number of in-between nodes set to be 1/2/3; the maximum length of the control paths set to be 2/3/4.

#### 2.6.3 Clinical trials

We checked which of the drugs identified in our analyses have been included in clinical trials for COVID-19. We looked for them first in the dedicated COVID-19 section of [8]. We then verified ClinicalTrails.gov [54] and IRTC [55] to identify drugs that weren’t listed yet on DrugBank. Of the 130 drugs we identified in our analyses, 23 have been investigated in clinical trials. We included in Supplementary Table 3, for each of the validated drugs, a selection of clinical trials dedicated to them. Of the 23 drugs included in clinical trials, 14 were obtained only on the MOI 0.01 network, 8 on both networks and one, ibuprofen, only on MOI 0.3 network. A discussion on some of these drugs is in Supplementary Information, Section 4.

For each of the drugs we identified, we included in Supplementary Table 4 a list of recent articles discussing their potential in COVID-19 therapies.

#### 2.6.4 Computational validation

More than half of the drugs identified by our analyses were also found through other computational approaches. Some of these drugs could have an inhibitory effect on the main protease (e.g. dabigatran etexilate [56, 57], dasatinib [58], ellagic acid [59, 60], radotinib [61]), on the papain-like protease (caffeine [62], phenformin [63], ximelagatran [64]) or on the RNA-dependent RNA polymerase (e.g. docetaxel [63], eribulin [65], nilotinib [66], pelitinib [67]), as well as on the interaction between the spike and ACE2 (e.g. dexibuprofen [68], midostaurin [69], paclitaxel [70], ponatinib [71], regorafenib [72]), or on another viral important point (e.g. nilotinib on nsp13 [73], isoprenaline on nsp9 [74], docetaxel on nsp14 [75], enzastaurin on nsp15 [76], entrectinib on nsp16 [77]). There are also drugs that can act on host and virus interactions (e.g. erlotinib [78], XL019 [6]) or only on host genes (e.g. lidocaine [79], kappadione [80, 81], phenethyl isothiocyanate [80]). As we can see, a drug can act on several points. For some of the drugs in our results we found some proposed theories on their potential mode of action (e.g. bryostatin [82], emodin [83–85] or ripretinib [86]). Others can be deduced based on their targets, identified in other studies (e.g. flovagatran, rimiducid, rindopepimut).

### 2.7 Robustness analysis

To test the robustness of the control analysis method, we performed additional analyses with variation in several of the parameters. For each MOI, we selected the sets consisting of the top 50, 100, 150, 200, 250 and 300 host factors and we synthesized the corresponding interaction networks while using 1, 2 and, respectively, 3 intermediate proteins for the interactions among the host factors and all known drug targets. We then ran the controllability analysis on each of the resulting networks, considering the maximum length of control paths 2, 3 and 4, for a total of 108 analyses. We aggregated the drugs identified in each individual analysis, and we compared the results obtained over all runs for each network and dataset. As it can be seen in Figure 5, on average, about 40% of the identified drugs for MOI 0.01 and about 25% for MOI 0.3 appear in over 60% of the corresponding runs.

## 3 Discussion

The recent study of [4] on the survivability of SARS-CoV-2-infected cells identified many host factors that are essential for the SARS-CoV-2 infection. This offers a new guide to therapeutic targeting of COVID-19. Yet, out of their top 200 ranked genes in each of the two viral load experiments only 23 (24, resp.) are drug targetable [8]. We investigated whether they can be instead targeted through short network signaling pathways and found 40 drug-targetable proteins that can control these genes. We identified 130 drugs that target these proteins and can be significant in COVID-19. Some of these include drugs recommended to be used, drugs that have been evaluated in clinical trials and various *in vitro* assays. The network-based approach offers a wider spectrum of options by identifying drug-targetable nodes at a short distance upstream of the SARS-CoV-2 infection host factors, able to influence them in the sense of network controllability. Moreover, we also identified in this way possible mechanisms of action for drugs, offering a mechanistic understandings of drugs through the changes they may induce in the PPIs.

Many of the drugs we identified act on the immune system (including the FDA-approved COVID-19 drug baricitinib) and on the coagulation cascade (e.g. nafamostat). Antivirals were not found by our analyses because for many of them (e.g. favipiravir, remdesivir, umifenovir) their human targets are unknown. Our list of drugs also includes false positives, such as drugs that are applied topically and should not be swallowed (e.g. ingenol mebutate), but also possible harmful substances in this disease (kappadione). They are found by our algorithms because they do influence the host factors, in addition to their other effects. Our results could be further improved by leveraging data on the quantitative strength of various interactions, on the result of their concurrent activation/inhibition signals and on the specificity of their mechanism in the context of the disease. Such data would be needed for all the PPIs in our network, not just for a selected subset of them. A quantitative version of network controllability theory remains to be developed to scale to become applicable to such data.

The network-based approach explored in this study can be applicable to other diseases and can be especially fruitful in drug repurposing for rare diseases. The key part of our approach is identifying a set of targets, whose control may be therapeutically beneficial. In this study we used as control targets the host factors required for SARS-CoV-2 infections. The network controllability analysis yields a set of input nodes that can control these targets. Moreover, the input nodes can be selected to a large extent to be drug targetable with currently available drugs, bringing this method within the drug repurposing realm. Each of the input nodes identified by the analysis (and the drugs targeting them), as well as various combinations of them, can be used to influence some of the control targets. This yields a rich set of predictions that could inform the setup of new drug repurposing clinical trials.

## 4 Materials and methods

### 4.1 Data

The signaling data were extracted from the KEGG [13], OmniPath [14] and SIGNOR [15] databases. Only the directed interactions were considered. An interaction can appear in multiple databases. The interactions were matched based on the UniProt identifiers of their proteins, which are provided by default by all of the three databases.

We considered as targets of our study the $200$ highest ranked host factors in the cell survivability experiments of [4] at 0.01 MOI and 0.3 MOI that were shown to be required for SARS-CoV-2 infection. These proteins were targeted through short multi-step signaling paths originating in drug targets. The drug targets were collected from the DrugBank [8] database. We selected the drug targets of the approved and investigational small molecule drugs, except for those illicit or nutraceutical. We also discarded a number of specific 19 approved and/or investigational drugs that have more than 50 targets, most of which are not targeted by any other drug.

### 4.2 Network generation

For each of the two MOI experiments of [4] we identified all proteins that are upstream of the top 200 highest ranked host factors, at a distance of maximum two interactions. We also identified all proteins that are downstream of the drug target proteins, at a distance of maximum two interactions. The network generation is discussed in all details in Supplementary Information, Section 1.

**Key Points**

- We constructed directed PPI networks linking drug-targetable nodes with the host factors required for the SARS-CoV-2 viral infection.
- Network controllability offers a new insight into the molecular details of COVID-19 and into potentially new therapies for it.
- We identified several drugs that have been investigated in clinical trials, but also some new ones, not yet studied in connection to COVID-19.
- The method is significant beyond COVID-19 and may be applied also to other diseases.

## Supplementary Material

supplementary_table_1_bbab490

supplementary_table_4_bbab490

## Data Availability

The drug target data set used in this article, the PPI networks and the code used to generate the networks, are available online in the GitHub repository [87].

## Notes

**Nicoleta Siminea** is a PhD student in Computer Science at the University of Bucharest, Romania and a research assistant at the National Institute of Research and Development for Biological Sciences, Romania. She is interested in finding new data-driven precision treatment opportunities with the help of bioinformatics methods.

**Victor Popescu** is a PhD student in Computer Science at Åbo Akademi University, Finland. His research interests include network science, computational systems biology, and software engineering.

**Jose Angel Sanchez Martin** is currently a postdoctoral researcher in the Department of Computer Science at the Technical University of Madrid, Spain. He has authored and co-authored several articles in high-impact factor journals. His research interests include natural computing, bioinformatics, precision medicine, deep learning and machine learning.

**Daniela Florea** is research assistant in the Department Bioinformatics, National Institute of Research and Development for Biological Sciences, Romania. Her research interests include fundamental and applied research, comparative determination of the toxicity of some nanoparticles synthesized for biomedical applications, the impact of nanoparticles on the environment and human health.

**Georgiana Gavril** is currently a researcher at the Department of Bioinformatics, National Institute of Research and Development for Biological Sciences, Romania. She has over 10 years of experience in scientific research in phytochemistry. Her research interests include deep learning, drug analysis in various diseases / conditions, cancer biology, computational biology.

**Ana-Maria Gheorghe** is currently a researcher at the National Institute of Research and Development for Biological Sciences, Romania. She has a Ph.D. in biology and has more than 10 years research experience. Her research interests include drug delivery systems with controlled and sustained delivery of therapeutic agents, wound dressings materials for skin chronic wound treatment and biotechnologies for transfusion medicine.

**Corina Iţcuş** is currently a research scientist in the Arctic and Antarctic Research Department at the National Institute of Research and Development for Biological Sciences, Romania. She has expertise in Genetics, Molecular Biology and Microbiology. Her research interests include DNA folding properties, microbial adaptations and diversity in extreme environments, cave microbiome and freshwater biology. She has authored and co-authored several top journal articles.

**Krishna Kanhaiya** is currently an affiliated researcher in the Department of Information Technologies, Åbo Akademi University, Turku, Finland. He has authored and co-authored several articles in high-impact factor journals. His research interests include bioinformatics, computational systems biology, network pharmacology, biomedical data analysis, bioinformatics database and pipelines construction, and cancer systems biology.

**Octavian Pacioglu** obtained his PhD in Environmental Sciences and is a freshwater ecologist with work experience in various types of habitats such as the hyporheic zone, caves, streams and deltas.

**Laura Ioana Popa** is a postdoctoral researcher with expertise and background in sequencing and bioinformatic analysis. Her research interests include antimicrobial resistance and DNA-modeling by using the DNA-Origami technique, a DNA-based nanotechnology method.

**Romica Trandafir** is Professor Emeritus (retired) at the Department of Mathematics and Computer Science, Technical University of Civil Engineering Bucharest, Romania. He is currently a researcher in the Department of Bioinformatics, National Institute of Research and Development for Biological Sciences, Bucharest, Romania. He has authored and co-authored several articles in Applied Mathematics, in Engineering, and in Bioinformatics. His research interests include modeling and simulation of some natural phenomena and multi-objective optimization problems.

**Maria Iris Tuşa** holds a PhD in Biology and works as a researcher at the National Institute of Research and Development for Biological Sciences, Romania. Her research interests include flow cytometry, molecular biology, environment microbiology.

**Manuela Sidoroff** is senior scientist in biotechnology at the National Institute of Research and Development for Biological Sciences, Romania. She has authored and co-authored more than 100 research papers, book chapters, conference papers and she holds four patents. Her research interests include analitycal and computational research, modeling of biotechnological processes, environmental technologies, and biodiversity. She coordinates the Romanian components of the pan-European project International Centre for Advances Studies River-Sea System ``DANUBIUS-RI'' (www.danubius-ri-.eu).

**Mihaela Păun** is currently a Senior Researcher at the National Institute of Research and Development for Biological Sciences, Romania and a Professor of Statistics at the University of Bucharest, Romania. She has more than 15 years' experience of teaching and research experience in the area of Bioinformation, Biocomputing and Biostatics. She has published more than 40 research papers in peer reviewed journals.

**Eugen Czeizler** is a senior researcher at the National Institute of Research and Development for Biological Sciences, Romania. His research is focused on algorithms and data analytics in bio-medicine, computational modelling, and algorithmic nanotechnology. The general goals of his research are to better understand the dynamics of complex bio-chemical and bio-medical systems and to develop optimal designs of synthetic systems performing well defined, large scale actions.

**Andrei Păun** is currently a Professor of Computer Science at University of Bucharest, Romania. He has published over 100 peer-reviewed journal publications, book chapters and conference papers. He has also two patents filed to his credit. His research interests include Biocomputing (P systems, DNA computing), Systems Biology and Automata Theory.

**Ion Petre** is Professor in the Department of Mathematics and Statistics, University of Turku, Finland, and a PI in the National Institute of Research and Development for Biological Sciences, Romania. His research interests are in computational systems biology, network science, data analytics, and theoretical computer science. He has published over 100 peer-reviewed journal publications, book chapters, and conference papers, and has edited 21 books and special issues of journals.
