# Supplementary material for: Network analytics for drug repurposing in COVID-19
Source: Brief Bioinform. 2021 Dec 7;23(1):bbab490. doi: 10.1093/bib/bbab490 (PMC8690228; doi:10.1093/bib/bbab490)
Supplement: supplementary_table_3_bbab490 [file supplementary_table_3_bbab490.pdf]

List of clinical trails found for each drug

- Acetylsalicylic acid
  - Acetylsalicylic Acid in the Prevention of Severe SARS-CoV2 Pneumonia in Hospitalised Patients With COVID-19 (Asperum) (phase 3, NCT04808895)
  - The LEAD COVID-19 Trial: Low-risk, Early Aspirin and Vitamin D to Reduce COVID-19 Hospitalizations (LEAD COVID-19) (phase 2, NCT04363840)
  - Protective Effect of Aspirin on COVID-19 Patients (PEAC) (phase 2/3, NCT04365309)
  - COVID-19 Positive Outpatient Thrombosis Prevention in Adults Aged 40-80 (NCT04498273, phase 3)
- Argatroban
  - Anticoagulation in Critically Ill Patients With COVID-19 (The IMPACT Trial) (IMPACT) (phase 4, NCT04406389)
- Arsenic trioxide
  - Efficacy of An Ayurvedic Preparation Raj Nirwan Bati (RNB) on symptomatic COVID-19 Patients (phase 3, CTRI/2020/06/025998)
- Baricitinib
  - Baricitinib Compared to Standard Therapy in Patients With COVID-19 (BARICIVID-19)(phase 2, NCT04393051)
  - Baricitinib Therapy in COVID-19 (phase 2/3, NCT04358614)
  - Clinical Trial to Evaluate Efficacy of 3 Types of Treatment in Patients With Pneumonia by COVID-19 (Covid19COVINIB) (phase 2, NCT04346147)
- Bivalirudin
  - Anticoagulation in Patients Suffering From COVID-19 Disease The ANTI-CO Trial (phase 4, NCT04445935)
- Dasatinib
  - Dasatinib for the Treatment of Moderate and Severe COVID-19 (phase 2, NCT04830735)
- Estradiol
  - The use of sex hormones in the control of coronavirus inflammation (phase 2/3, IRCT20150716023235N15)
  - Estrogen Patch for COVID-19 Symptoms (phase 2, NCT04359329)
- Estradiol cypionate
  - Estradiol and Progesterone in Hospitalized COVID-19 Patients (phase 2, NCT04865029)
- Genistein
  - BIO 300 Oral Suspension in Discharged COVID-19 Patients (phase 2, NCT04482595)
- Ibuprofen
  - LIBERATE Trial in COVID-19 (LIBERATE) (phase 4 , NCT04334629)
  - Inhaled Ibuprofen to Treat COVID-19 (CórdobaTrail) (phase - not applicable, NCT04382768)
- Imatinib
  - Clinical Trial to Evaluate Efficacy of 3 Types of Treatment in Patients With Pneumonia by COVID-19 (Covid19COVINIB) (phase 2, NCT04346147)

- The Safety & Efficacy of Imatinib for the Treatment of SARS-COV-2 Induced Pneumonia (phase 3, NCT04422678)
  - Trial of Imatinib for Hospitalized Adults With COVID-19 (phase 3, NCT04394416)

- Lidocaine
  - Impact of Intravenous Lidocaine on Clinical Outcomes of Patients With ARDS During COVID-19 Pandemia (LidoCovid)) (phase 3, NCT04609865)

- Lithium carbonate
  - A study of drug Lithium on patients of Covid 19 disease (phase - not available, CTRI/2020/06/026193)
  - Clinical trial of lithium in improving the clinical and laboratory symptoms of patients with COVID-19 (phase 2, IRCT20081019001369N5)
  - Comparative assessment of the efficacy and safety of addon treatment with “Sofosbuvir-Daclatasvir”, “Lithium”,and “Trifluoprazine” to “standard of care in three groups of patients with COVID-19 (phase 3, IRCT20130812014333N147)

- Minocycline
  - Clinical trial of minocycline against COVID-19 (phase 2, IRCT20081019001369N4)

- Nafamostat
  - A study to evaluate the efficacy and safety of Nafamostat Mesilate in treatment of Coronavirus infection (phase 2, CTRI/2020/06/026220)
  - Clinical Efficacy of Nafamostat Mesylate for COVID-19 Pneumonia (phase 2/3, NCT04418128)
  - Efficacy of Nafamostat in Covid-19 Patients (RACONA Study) (RACONA)(phase 2/3, NCT04352400)

- Quercetin
  - Effect of Quercetin on Prophylaxis and Treatment of COVID-19 (phase-not applicable , NCT04377789)
  - The Study of Quadruple Therapy Zinc, Quercetin, Bromelain and Vitamin C on the Clinical Outcomes of Patients Infected With COVID-19 (phase 4, NCT04468139)
  - Evaluation of the effect of quercetin on the effectiveness of antiviral drug regimen in patients with COVID19 (phase 3, IRCT20200419047128N2)

- Resveratrol
  - Evaluation efficacy of "Curcumin and Resveratrol" capsule in controlling symptoms in patients with COVID-19 (phase 3, IRCT20080901001165N56)
  - Evaluation of the effect of Resveratrol on the effectiveness of antiviral drug regimen in patients with COVID-19 (phase 3, IRCT20200112046089N1)
  - Randomized Proof-of-Concept Trial to Evaluate the Safety and Explore the Effectiveness of Resveratrol, a Plant Polyphenol, for COVID-19 (phase 2, NCT04400890)
  - The Anti-fibrotic Therapeutic Effects of Resveratrol for Discharged COVID-19 Patients (HK-COVID19Res) (phase - not applicable, NCT04799743)
  - Can SARS-CoV-2 Viral Load and COVID-19 Disease Severity be Reduced by Resveratrol-assisted Zinc Therapy (Reszinate) (phase 2, NCT04542993)

- Ruxolitinib
  - Ruxolitinib for Acute Respiratory Disorder Syndrome Due to COVID-19 (RUXO-COVID) (phase 2/3, NCT04477993)
  - Ruxolitinib for Treatment of Covid-19 Induced Lung Injury ARDS (RuXoCoil) (phase 2, NCT04359290)

- Sirolimus
  - Sirolimus Treatment in Hospitalized Patients With COVID-19 Pneumonia (SCOPE) (phase 2, NCT04341675)
  - Efficacy and Safety of Sirolimus in COVID-19 Infection (phase 2, NCT04461340)
  - Effects of mTOR Inhibition With Sirolimus (RAPA) in Patients With COVID-19 to Moderate the Progression of ARDS (RAPA-CARDS) (phase 1/2, NCT04482712)
- Suramin
  - A multi-center study on the efficacy and safety of suramin sodium in adult patients with novel coronavirus pneumonia (COVID-19) (phase 0, ChiCTR2000030029)
- Tamoxifen
  - Combination Therapy With Isotretinoin and Tamoxifen Expected to Provide Complete Protection Against Severe Acute Respiratory Syndrome Coronavirus (Combination) (phase 2, NCT04389580)
  - Combination of Chemopreventive Agents (All- Trans Retinoic Acid and Tamoxifen) as Potential Treatment for the Lung Complication of COVID-19 (phase 2, NCT04568096)
- Tofacitinib
  - Tofacitinib in Hospitalized Patients With COVID-19 Pneumonia (phase 2, NCT04469114)
  - Tofacitinib for Treatment of Moderate COVID-19 (I-TOMIC) (phase 2, NCT04415151)
- Zanubrutinib
  - Covid-19 Infection and Pulmonary Distress Treatment With Zanubrutinib in Hospitalized Participants (phase 2, NCT04382586)
