# Supplementary material for: Network analytics for drug repurposing in COVID-19
Source: Brief Bioinform. 2021 Dec 7;23(1):bbab490. doi: 10.1093/bib/bbab490 (PMC8690228; doi:10.1093/bib/bbab490)
Supplement: supplementary_table_4_bbab490 [file supplementary_table_4_bbab490.pdf]

List of scientific articles found for each drug

- Acetylsalicylic acid
  - may reduce the incidence of COVID-19-induced coagulopathy [1]
  - an analysis of 232 patients, including 204 who did not take aspirin and 28 who did not take it, found that low-dose aspirin was associated with a lower risk of mortality [2]
- Afatinib
  - potential drug based on a silico study [3]
  - potential protease inhibitor potential based on virtual screening [4]
  - candidate drug based on a protein interaction map [5]
  - drug repurposed by probabilistic networks and crowdsourced curation [6]
  - one of the top scores obtained by AI method [7]
- Alvocidib
  - up-regulates the 50 genes most correlated with ACE2 based on RNAseq (machine learning methods) [8]
  - may affect the virus throught CDK9 (expert review) [9]
  - may have antiviral properties according to a signature-based approach [10]
  - inhibited the formation of cytopathic effect induced by virus infection in Vero E6 cells at 1 μM, but was also toxic to the cell [11]
  - drug that significantly altered the expression of genes resulted from an unsupervised method (in silico) [12]
- Argatroban
  - 10 patients who were resistant to heparinization were anticoagulated with argatroban [13]
  - can be used for anticoagulation in case of heparin resistance (two cases reported) [14]
  - alteplase and argatroban in massive pulmonary embolism (one case reported) [15]
- Arsenic trioxide
  - may block most of the viral infection pathways according to an in silico study [16]
  - as a treatment proposal for the fibrogenic effect of TGF - β [17]
- AT-7519
  - drug obtained on the basis of a network approach [5]
  - one of the three top-scored repurposed drugs resulted from a docking study [18]
- Baricitinib
  - can act on the cytokine storm and on the intracellular passage and assembly of SARS-CoV-2 in target cells [19]
  - suppresses the production of proinflammatory cytokines, preserves innate antiviral responses and SARS-CoV-2 T cells, and limits the recruitment of neutrophils to the lungs and neutrophil cell death (NETosis)[20]
  - in a study of 62 patients who received baricitinib and coticosteroids and 50 patients who received corticosteroids, an improvement in respiratory function was observed [21]
  - may block the JAK-STAT signal leading to an impairment of the interferon-mediated antiviral response and therefore to a possible facility of the virus [22]

- on a group of 20 patients, prevented progression to a severe form by modulating the patient's immune landscape [23]
  - significant increase in virus growth in an in vitro assay [24]

- Bivalirudin
  - as a direct thrombin inhibitor, may decrease the action of thrombin in circulating and clot-related sites [25]
  - can be used in COVID-19 due to the rapid achievement of therapeutic anticoagulation and stable pharmacokinetics [26]

- Bosutinib
  - it should be used in COVID-19 as it is a powerful anti-inflammatory agent [27]
  - could be investigated for SARS-CoV-2 infection because it appears to have some antiviral activity in vitro [28]
  - may inhibit SARS-CoV-2 replication [29]

- Brigatinib
  - drug repurposed using probabilistic networks and crowdsourced curation [6]
  - EGFR inhibitors as potential COVID-19 drugs [30]

- Bryostatin 1
  - potential anti-coronavirus therapy (review) [31]

- Caffeine
  - can block ACE2 exposure to the spike protein (in silico study) [32]
  - potential inhibitor against 3CLpro according to an in silico study [33]

- Canertinib
  - drug that significantly altered the expression of genes resulted from an unsupervised method [12]
  - EGFR inhibitors as potential COVID-19 drugs [30]
  - can target MASP-2, hyperactivation of which has been observed in patients with coronaviruses [34]

- Cefazolin
  - could be repurposed as effective drug based a machine learning method [35]
  - can be used in bacterial suprainfection [36]

- Cholecystokinin
  - endosomal acidification depends on endosomal Ca2+ release and CCK mobilizes intracellular Ca2+ [37]

- Dabigatran etexilate
  - potential RNA-dependent RNA polymerase inhibitor [38]
  - could be active against main protease (in silico studies) [39], [40]

- Dabrafenib
  - can inhibit SARS-CoV-2 infection [41]

  - is predicted to inhibit human protein NEK9, which is an interactor for the SARS-CoV-2 nsp9 protein [42]
  - significant increase in virus growth in an in vitro assay [24]
  - could have an impact on SARS-CoV-2 biogenesis [43]

- Dacomitinib
  - is active in SARS-CoV-2 infection across diverse cell types [44]
  - may target GRP78, a gene that has been shown to be 4 times higher in patients with COVID-19 [45]
  - potential covalent inhibitor for cathepsin B/L [46]

- Dasatinib
  - it can be active on SARS-CoV-2 as it is active in vitro on MERS and SARS [47], [48]
  - one of the most potent inhibitors of the SARS-CoV-2 3CL protease - computational study [49]

- Dexibuprofen
  - molecular docking identifies dexibuprofen as ligand for spike protein [50]
  - drug identified based on host transcriptome [51]

- Docetaxel
  - showed a strong affinity for RNA-dependent RNA polymerase [52]
  - possible binder for SARS-CoV-2 nsp14 protein, weaker than ritonavir [53]
  - can block spike SARS-CoV-2 interaction with ACE2 (in vitro) [54]

- Doramapimod
  - inhibited SARS-CoV-2 replication in vitro [55]
  - may act on modified phosphorylation from SARS-CoV-2 infection [56]

- Ellagic acid
  - may inhibit the function of the SARS-CoV2 E protein and may reduce inflammation and may have a protective role in endotoxin-induced septic shock [57]
  - showed significant binding interactions with SARS-CoV-2 receptor-binding domain, 3CLpro and GRP78 (in silico) [58]
  - the highest binding affinities against nsp9 and nsp10 according to quantum chemical, molecular docking and dynamic methods [59]
  - showed significant binding to the catalytic site of the Mpro enzyme [60]
  - inhibitor of SARS-CoV-2 Mpro due to its excellent binding energies [61]

- Emodin
  - may interfere with SARS S interaction to ACE2 [62], so it should be tested [63], [64], [65]

- Encorafenib
  - potential compound for the treatment of COVID-19 because it has a high affinity for the main protease, but also has side effects [66]

- Entrectinib
  - potential inhibitor of nsp16 2'-O-ribose methyltransferase of SARS-CoV-2 [67], [68]
  - may bind to SARS-CoV-2 receptor-binding domain [69]

  - could inhibit SARS-CoV-2 infection based on a machine learning approach [70]
  - non-selective bradykinin receptor antagonist based on an in silico study, therefore could have an effect in inflammation associated with the disease [71]
  - can block spike SARS-CoV-2 interaction with ACE2 (in vitro) [54]

- Enzastaurin
  - may act on modified phosphorylation from SARS-CoV-2 infection [56]
  - potential inhibitor for SARS-CoV-2 endoribonuclease [72]

- Eribulin
  - one of 22 compounds resulting after virtual screening of the FDA approved drugs against the 2,-O-methyltransferase, but which was not in the top 5 after re-docking [73]
  - possess similar pharmacophore features of main protease inhibitors [74]
  - can target RNA-dependent RNA polymerase as it can target human telomerase reverse transcriptase which has phylogenetic and structural similarities to viral RdRPs [75]

- Erlotinib
  - can inhibit SARS-CoV and SARS-CoV-2 at 10 μM [76]
  - can reduce the infectivity of a wide range of viruses and is also an EGFR inhibitor, so it can reduce cardiovascular complications as the involvement of EGFR transactivation in these complications has been observed [77]
  - drug repurposing candidate based on host-drug interactome [78]

- Estradiol
  - is involved in the body's immune response, attenuates the vasoconstrictor response to various stimuli and induces vasodilation in hypoxia [79]
  - drug repurposed based on an in silico study [80]
  - decreases COVID-19 fatality according to an analysis of 68,466 cases [81]
  - may increase the expression/activity of ACE2 in the adipose tissue and the kidney [82]

- Estradiol benzoate
  - the most favorable option for targeting the host entry CD147 protein based on virtual screening and simulation of molecular dynamics [83]
  - may inhibit the viral entry of SARS-CoV-2 as it blocks protein-mediated membrane fusion [84]
  - can block spike SARS-CoV-2 interaction with ACE2 (in vitro) [54]

- Estradiol cypionate
  - predicted drug based on a network approach [85]

- Estradiol valerate
  - potential 3CLpro inhibitor [38]

- Everolimus
  - a rapamycin derivative that should be investigated for COVID-19 [86]
  - an mTOR inhibitor may be useful in COVID-19 [87]
  - inhibition of mTORC1 can be useful in COVID-19 [88]
  - can block spike SARS-CoV-2 interaction with ACE2 (in vitro) [54]

- Fasudil
  - may help in associated acute lung injury and acute respiratory distress syndrome [89]
  - up-regulate ACE2 expression and down regulate ACE expression[90]
  - rho-kinase inhibitors are potential drugs in SARS-CoV-2 infection [91]
  - has a protective effect against lung damage, and possesses antifibrotic action and the ability to up-regulate ACE2[92]
  - can improve vasodilatation and ACE2 activity [93]
- Fedratinib
  - may reduce mortality of patients with TH17 type immune profiles [94]
  - JAK-STAT inhibitors may have promising effects in SARS-CoV-2 infection [95]
- Gabexate
  - could inhibit SARS-CoV-2 infection as it targets TMPRSS2 [96]
  - is an inhibitor of complementary pathways and a broad-spectrum anti-inflammatory agent [77]
  - inactive on SARS-CoV-2 S protein fusion in the range of tested concentrations (10 nM-10 μM) [97]
  - SARS-CoV-2 S host cell entry is slightly inhibited [98]
- Gefitinib
  - anti-GFRs drugs against COVID-19 (hypothesis) [99]
- Genistein
  - can block GRP78 SBDβ and thus could be explored to inhibit SARS-CoV-2 infection [100]
  - may limit viral spread because it interacts with the binding domain of the spike [101]
- Ibuprofen
  - potential treatment against SARS-CoV-2 Mpro [102]
  - may be useful in citokine storm [103]
- Icotinib
  - EGFR inhibitors as potential COVID-19 drugs (in silico) [30]
- Imatinib
  - is not a potent anti-SARS-CoV-2 drug because it has no in vitro activity on SARS-CoV-2 replication (tested in human Cacao-2 cells) [104]
  - antiviral and immunomodulatory agent that could be an option for COVID-19 pneumonia[105]
  - suppress SARS-CoV-2 replication on Vero E6 cells [106]
  - entry inhibitor of SARS-CoV-2 on a lung organoid model using human pluripotent stem cells[107]
  - it is active on MERS and SARS and it can be active on SARS-CoV-2 [48], [108], [109]
  - presented inhibitory activity in Vero E6 cells [110]
- Ingenol mebutate
  - ingenol is one of the six possible broad-spectrum inhibitors that are active on SARS and MERS pseudotyped particles entry [111]

- Isoprenaline
  - one of the top-ranked hits for SARS-CoV-2 nsp9 [112]
- Kappadione
  - predicted drug based on SARS-CoV-2 infected host interactome [16]
  - strong interaction with RdRp [113]
  - resulted from the network analysis of the genes involved in the host response [114]
- Lapatinib
  - inhibited SARS-CoV-2 replication in vitro [55]
  - suppresses replication of SARS-CoV-2 and unrelated viruses [115]
- Lidocaine
  - has anti-inflammatory properties and may be useful in tempering the cytokine storm [116]
  - could be beneficial in reducing cytokines, protecting patients' lungs and improving outcomes in patients with COVID-19 [117]
  - proposed as COVID-19 based on transcriptomic profiling of SARS-CoV-2 infection [118]
- Lithium carbonate
  - in a study of six patients with severe COVID-19 infection, it improved both inflammatory activity and the immune response [119]
  - could mitigate NLRP3-mediated cytokine storm (hypothesis) [120]
  - potential drug based on machine learning techniques [121]
- Lithium citrate
  - drug repurposed based on machine learning techniques [121]
- Midostaurin
  - may act on modified phosphorylation from SARS-CoV-2 infection [56]
  - could be a spike protein – ACE2 interaction inhibitor based on an in silico study [122]
  - one the results of the virtual screening of approved drugs as potential SARS-CoV-2 main protease inhibitors [123]
  - agonist/test inconclusive [124]
  - activates viral infection in vitro[42]
- Minocycline
  - there are several hypotheses such as that it may have antiviral properties and may be useful in the citokine storm [125], [126] and it can counteract the neurological impact [127]
  - azithromycin should be replaced with minocycline because it has the same effect but with a reduced arrhythmogenic effect [128]
  - predicted as a potent inhibitor of main protease [129]
- Nafamostat
  - inhibited mediated entry into host cells with an efficiency approximately 15 times higher than camostat mesylate [98]
  - blocked infection of Calu-3 cells with an effective concentration (EC) 50 around 10 nM, while a significantly higher dose (EC50 around 30 μM) was required for VeroE6 / TMPRSS2 [97]

  - nafamostat mesylate therapy in combination with favipiravir may block the entry and replication of the virus as well as inhibit hypercoagulopathy (a series of 11 cases) [130]
  - was the most potent drug for human lung cells (IC50 = 0.0022 μM, of the 24 drugs selected [131]

- Navitoclax
  - inactive on main protease according to the modelling of quantitative structure-activity relationship [132]

- Neflamapimod
  - may act on modified phosphorylation from SARS-CoV-2 infection [56]

- Neratinib
  - main protease inhibitor based on a computational identification [133]
  - potential covalent inhibitor for cathepsin B/L [46]
  - may target GRP78, a gene that has been shown to be 4 times higher in patients with COVID-19 [45]

- Netarsudil
  - rho-kinase inhibitors could be an option in SARS-CoV-2 infection [91]

- Nilotinib
  - may inhibit SARS-CoV-2 in vitro (Vero-E6 cells and Calu-3 cells [134]
  - one of the eight compounds obtained on the basis of virtual screening of potential inhibitors targeting RNA-dependent RNA polymerase activity (NSP12) [135]
  - has anti SARS-CoV-2 activity [136]
  - has been predicted to bind to the receptor-binding domain of SARS-CoV-2 spike protein [137]
  - inhibitory effect on SARS-CoV-2 nsp13 [138]
  - has anti -SARS-CoV-2 effects by inhibiting DDX42 protein [139]
  - can block spike SARS-CoV-2 interaction with ACE2 (in vitro) [54]

- Obatoclax
  - can inhibit SARS-Cov-2 replication in vitro [140]
  - has antiviral properties in Vero-E6 cells [141]

- Olmutinib
  - main protease inhibitor (computational study) [142]
  - EGFR inhibitors as potential COVID-19 drugs (in silico) [30]
  - can block spike SARS-CoV-2 interaction with ACE2 (in vitro) [54]

- Osimertinib
  - is one of the six possible broad-spectrum inhibitors that are active on SARS and MERS pseudotyped particles entry [111]
  - has the potential to be repurposed as SARS-CoV-2 drug based on its potency, pharmacokinetic and human safety profile [143]

- Paclitaxel
  - can increase cellular methylglyoxal concentration to virucidal levels [144]
  - affects the production of infectious extracellular virus [145]

- may target an extremely conserved domain of SARS-CoV-2 Spike [146]

- Pelitinib
  - one of the 10 best-docked compounds against SARS-CoV-2 RdRp [147]
  - potent antiviral activity and low cytotoxicity [148]
  - EGFR inhibitors to treat severe or risk cases [30]
- PH-797804
  - may act on modified phosphorylation from SARS-CoV-2 infection [56]
  - can be used to limit lung injury [149]
- Phenethyl Isothiocyanate
  - drug identified using proteome-based SARS-CoV-2 infected host biology [16]
  - can inhibit Mpro in a dose dependent manner [150]
  - may be active against 3CLpro and PLpro, but inhibition is incomplete [151]
- Phenformin
  - has binding affinities for COVID-19 Plpro [52]
- Pimecrolimus
  - may inhibit SARS-CoV-2 main protease, 3CLpro [152]
- Ponatinib
  - may inhibit SARS-CoV-2 replication by non-specific effects (off-target) [106]
  - may target GRP78, a gene that has been shown to be 4 times higher in patients with COVID-19 [45]
  - RIPK1, which plays some roles in apoptosis, necroptosis and inflammatory pathways, is thought to be inhibited by this drug but did not have the desired in vitro effect at high doses [42]
  - was active in viral inhibition in Paris [124]
  - has affinity for the SARS-CoV-2 S-protein S1 subunit, which contains the receptor binding domain [153]
  - possible active drug obtained through a network medicine framework [154]
- Proflavine
  - proflavine inhibits PLpro [155]
- Pseudoephedrine
  - blocker for SARS-CoV-2 spike pseudovirus entering the ACE2 over-expressed HEK293T cell line [156]
- Quercetin
  - possible active drug obtained through a network medicine framework [154]
  - may inhibit SARS-CoV-2 furin enzyme and spiked glycoprotein based on a computational study [157]
  - could help in COVID-19-induced acute kidney injury (network pharmacology and molecular docking study) [158]
  - inhibit SARS-CoV-2-Mpro [159]

- could help in COVID-19 because it is an anti-inflammatory, antioxidant, analgesic and inflammatory compound [160]
- potent inhibitor resulting from an experimental in vitro molecular screening procedure [161]
- dug repurposed based on an in silico study [80]

- Radotinib
  - main protease inhibitor based on a virtual screening [4]
- Rasagiline
  - drug possible active based on Bayesian networks on proteomic dataset generated from Caco-2 cells transfected with SARS-CoV-2 [162]
- Regorafenib
  - increased SARS-CoV-2 growth in an in vitro infection assay [24]
  - one of the 33 compounds obtained as potential inhibitors of Mpro [163]
  - possible role in the receptor mediated host response to SARS-Cov-2 [164]
  - potential effect on the viral receptor-binding domain spike S1–ACE2 interface [165]
- Resveratrol
  - can be used in COVID-19 as it can interfere with spike-ACE2 according to a docking study [166]
  - antioxidant and has antiviral effect against several viruses [167]
  - can up-regulate ACE2, so as to have a protective effect on SARS-CoV-2 illness severity [168]
  - has strong antiviral properties against SARS-CoV-2 in vitro [169]
  - inhibits replication in Vero cells [170]
- Ridaforolimus
  - a rapamycin derivative that should be investigated for COVID-19 [86]
- Ripretinib
  - diarylureas possess antiviral, antithrombotic and anti-inflammatory properties that may be useful in COVID-19 [171]
- Ruxolitinib
  - combination of ruxolitinib and eculizumab in a study of 17 patients showed improvements in respiratory symptoms, radiographic lung damage and decreased circulating levels of dimer D [172]
  - can be used in systemic hyperinflammation [173]
  - can be used in SARS-CoV-2 infection associated with ARDS (a case study) [174]
  - low dose of ruxolitinib plus steroids could be used in severe SARS-CoV-2 pneumonia based on a non-randomized clinical trial [175]
  - an important potential in overcoming the complications caused by the immune hyperactivation related to the JAK / STAT signaling pathway [176]
  - showed efficacy in a pilot cases series in patients with severe COVID-19 [177]
  - can be used in severe cases based on a randomised trial [178]
- Seliciclib
  - drug identified by key host pathways asociated with SARS-CoV-2 infection [16]

  - may help with SARS-CoV-2 infection as it may act on T cell and NK cell apoptosis[179]

- SF1126
  - can inhibit the activity of DNA-dependent protein kinase which is a key factor in SARS-CoV-2 lifecycle [180]
- Sirolimus
  - may act on modified phosphorylation from SARS-CoV-2 infection [56]
  - may target an extremely conserved domain of SARS-CoV-2 Spike [146]
  - drug repurposed based on a HCoV–host interactome [181]
  - it is possible to restore T-cell functionality and to decrease cytokine storm [182]
  - may be repurposed for COVID-19 [183]
  - may be useful in COVID-19 because it is an mTOR inhibitor [87]
  - may be useful in COVID-19 according to a network analysis [184]
  - inhibition of mTORC1 can be useful in COVID-19 [88]
- Sorafenib
  - may act on modified phosphorylation from SARS-CoV-2 infection [56]
  - prevents SARS-CoV-2 replication in vitro [185]
  - drug that significantly altered the expression of genes resulted from an unsupervised method (in silico) [12]
  - may increase SARS-CoV-2 growth in vitro [24]
  - the cytotoxic and antiviral IC50 values are close [164]
- Sucralfate
  - coluld help to prevent further transmission from the stomach to the intestine [186]
  - has a potential binding affinity with spike protein [187]
- Suramin
  - may interfere with the onset of infection according to cell culture tests [188]
  - inhibitor of SARS-CoV-2 3CLpro [189], [190]
- Talmapimod
  - may act on modified phosphorylation from SARS-CoV-2 infection [56]
  - a kinase inhibitor active in SARS-CoV-2 infection [164]
- Tamoxifen
  - in vitro, it showed a more than 100-fold reduction in viral load[191]
  - presented inhibitory activity in Vero E6 cells [110]
  - should be tested because it is a calmodulin antagonist, therefore inhibits the CALM-ACE2 interaction and increases the release of the ACE2 ectodomain [192]
- Tapinarof
  - one of 30 drugs obtained that can be repurposed based on network bioinformatics analyses [193]
- Temsirolimus
  - could interact whith 3CLpro [152]

  - can block spike SARS-CoV-2 interaction with ACE2 (in vitro) [54]
- Tesevatinib
  - EGFR inhibitors as potential COVID-19 drugs [30]
- Tideglusib
  - SARS-CoV-2 main protease inhibitor [194],[195], [196]
- Tiludronic acid
  - one of the highest score based on molecular docking of the SARS-CoV-2 Spike protein [197]
- Tofacitinib
  - JAK inhibitors can be used to limit inflammation according to a a systematic review and meta-analysis [198]
- Ulixertinib
  - ameliorated SARS-CoV-2 spike protein subunit 1-induced inflammation as well as epithelial damage in vitro [199]
- Vandetanib
  - could inhibit main protease activity [3]
  - drug repurposed by probabilistic networks and crowdsourced curation [6]
- Varlitinib
  - potential anti-SARS-CoV-2 drug obtained through virtual screenings [200]
  - EGFR inhibitors as potential COVID-19 drugs [30]
- Vemurafenib
  - identified based on a protein interaction map [5]
  - can target nucleotide binding domain of the protein to which the virus binds [201]
- Venetoclax
  - obtained on the basis of virtual drug screening on the active sites of SARS-CoV-2 3CL pro [202]
- Voclosporin
  - possibly useful in infection according to a mechanism-driven neural network-based method [203]
- VX-702
  - may act on modified phosphorylation from SARS-CoV-2 infection [56]
- Ximelagatran
  - can interact with COVID-19 papain-like protease according to a docking study [204]
  - drug repurposed based on a network algorithm [205]
  - potential RNA polymerase inhibitor (docking study) [206]
- XL019
  - drug repurposed obtained on the basis of a network containing all interactions involving SARS-CoV-2 proteins, human interaction proteins, diseases and symptoms [207]
- Zanubrutinib
  - it can be used for its anti-inflammatory effects in COVID-19 [31]
  - it decreases levels of pro-inflammatory cytokines that are commonly elevated in severe COVID-19 [208]

References

[1] M. Gavillet, D. L. Rolnik, M. K. Hoffman, A. Panchaud, and D. Baud. Should we stop aspirin prophylaxis in pregnant women diagnosed with COVID-19? *Ultrasound in Obstetrics & Gynecology*, 55(6):843–844, 2020.

[2] Qiang Liu, Na Huang, Anni Li, Yuanhong Zhou, Liang Liang, Xinyu Song, Zhanqiu Yang, and Xiaolin Zhou. Effect of low-dose aspirin on mortality and viral duration of the hospitalized adults with COVID-19. *Medicine*, 100(6), 2021.

[3] Arun Gangadharan, Chelankara Sharanya, Abhithaj Jayanandan, and Chittalakkottu Sadasivan. Drug repurposing to identify therapeutics against COVID 19 with SARS-Cov-2 spike glycoprotein and main protease as targets: An in silico study. *chemrxiv*, April 2020.

[4] Olujide O. Olubiyi, Maryam Olagunju, Monika Keutmann, Jennifer Loschwitz, and Birgit Strodel. High throughput virtual screening to discover inhibitors of the main protease of the coronavirus SARS-CoV-2. *Molecules*, 25(14), 2020.

[5] Claudia Cava, Gloria Bertoli, and Isabella Castiglioni. A protein interaction map identifies existing drugs targeting SARS-CoV-2. *BMC Pharmacology and Toxicology*, 21(1):65, 2020.

[6] David J. Skelton, Aoesha Alsobhe, Elisa Anastasi, Christian Atallah, Jasmine E. Bird, Bradley Brown, Dwayne Didon, Phoenix Gater, Katherine James, David D. Lennon Jr au2, James McLaughlin, Pollyanna E. J. Moreland, Matthew Pocock, Caroline J. Whitaker, and Anil Wipat. Drug repurposing prediction for COVID-19 using probabilistic networks and crowdsourced curation. *arXiv preprint arXiv:2005.11088*, 2020.

[7] Konstantin Avchaciov, Olga Burmistrova, and Peter Fedichev. Ai for the repurposing of approved or investigational drugs against COVID-19, March 2020.

[8] Maxim V. Kuleshov, Daniel J. Stein, Daniel J.B. Clarke, Eryk Kropiwnicki, Kathleen M. Jagodnik, Alon Bartal, John E. Evangelista, Jason Hom, Minxuan Cheng, Allison Bailey, Abigail Zhou, Laura B. Ferguson, Alexander Lachmann, and Avi Ma'ayan. The COVID-19 drug and gene set library. *Patterns*, 1(6):100090, 2020.

[9] Ellen Weisberg, Alexander Parent, Priscilla L. Yang, Martin Sattler, Qingsong Liu, Qingwang Liu, Jinhua Wang, Chengcheng Meng, Sara J. Buhrlage, Nathanael Gray, and James D. Griffin. Repurposing of kinase inhibitors for treatment of COVID-19. *Pharmaceutical Research*, 37(9):167, 2020.

[10] Sinead M. O'Donovan, Ali Imami, Hunter Eby, Nicholas D. Henkel, Justin Fortune Creeden, Sophie Asah, Xiaolu Zhang, Xiaojun Wu, Rawan Alnafisah, R. Travis Taylor, James Reigle, Alexander Thorman, Behrouz Shamsaei, Jarek Meller, and Robert E. McCullumsmith. Identification of candidate repurposable drugs to combat COVID-19 using a signature-based approach. *Scientific Reports*, 11(1):4495, 2021.

[11] Jing Xing, Rama Shankar, Aleksandra Drelich, Shreya Paithankar, Evgenii Chekalin, Thomas Dexheimer, Mei-Sze Chua, Surender Rajasekaran, Chien-Te Kent Tseng, and Bin Chen. Analysis of infected host gene expression reveals repurposed drug candidates and time-dependent host response dynamics for COVID-19. *bioRxiv*, 2020.

[12] Y.-h. Taguchi and Turki Turki. A new advanced in silico drug discovery method for novel coronavirus (SARS-CoV-2) with tensor decomposition-based unsupervised feature extraction. *PLOS ONE*, 15(9):e0238907, September 2020.

[13] Deepa J. Arachchillage, Christopher Remmington, Alex Rosenberg, Tina Xu, Maurizio Passariello, Donna Hall, Mike Laffan, and Brijesh V. Patel. Anticoagulation with argatroban in patients with acute antithrombin deficiency in severe COVID-19. *British Journal of Haematology*, 190(5):e286–e288, 2020.

[14] Fergal McGlynn, Jennifer McGrath, Chithra Varghese, Barbara Ryan, Johnny McHugh, Arabella Fahy, and Helen Enright. Argatroban for therapeutic anticoagulation for heparin resistance associated with covid-19 infection. *Journal of Thrombosis and Thrombolysis*, 51(1):243–245, 2021.

[15] Lisa M Sagardia and Lisa M Daniels. Thrombolysis and use of argatroban for the treatment of massive pulmonary embolism following anticoagulation failure in a patient with COVID-19. *American Journal of Health-System Pharmacy*, 77(23):1961–1964, 08 2020.

[16] Debmalya Barh, Sandeep Tiwari, Marianna E. Weener, Vasco Azevedo, Aristóteles Góes-Neto, M. Michael Gromiha, and Preetam Ghosh. Multi-omics-based identification of SARS-CoV-2 infection biology and candidate drugs against COVID-19. *Computers in Biology and Medicine*, 126:104051, 2020.

[17] Pengfei Sun, Shuyan Qie, Zongjian Liu, Jizhen Ren, Kun Li, and Jianing Xi. Clinical characteristics of hospitalized patients with SARS-CoV-2 infection: A single arm meta-analysis. *Journal of Medical Virology*, 92(6):612–617, 2020.

[18] Tania Islam, Md Rezanur Rahman, Busra Aydin, Hande Beklen, Kazim Yalcin Arga, and Md Shahjaman. Integrative transcriptomics analysis of lung epithelial cells and identification of repurposable drug candidates for COVID-19. *European Journal of Pharmacology*, 887:173594, 2020.

[19] Xiuhong Zhang, Yan Zhang, Weizhen Qiao, Ji Zhang, and Zhigang Qi. Baricitinib, a drug with potential effect to prevent sars-cov-2 from entering target cells and control cytokine storm induced by COVID-19. *International immunopharmacology*, 86(32645632):106749–106749, September 2020.

[20] Timothy N. Hoang, Maria Pino, Arun K. Boddapati, Elise G. Viox, Carly E. Starke, Amit A. Upadhyay, Sanjeev Gumber, Michael Nekorchuk, Kathleen Busman-Sahay, Zachary Strongin, Justin L. Harper, Gregory K. Tharp, Kathryn L. Pellegrini, Shannon Kirejczyk, Keivan Zandi, Sijia Tao, Tristan R. Horton, Elizabeth N. Beagle, Ernestine A. Mahar, Michelle Y. H. Lee, Joyce Cohen, Sherrie M. Jean, Jennifer S. Wood, Fawn Connor-Stroud, Rachelle L. Stammen, Olivia M. Delmas, Shelly Wang, Kimberly A. Cooney, Michael N. Sayegh, Lanfang Wang, Peter D. Filev, Daniela Weiskopf, Guido Silvestri, Jesse Waggoner, Anne Piantadosi, Sudhir P. Kasturi, Hilmi Al-Shakhshir, Susan P. Ribeiro, Rafick P. Sekaly, Rebecca D. Levit, Jacob D. Estes, Thomas H. Vanderford, Raymond F. Schinazi, Steven E. Bosinger, and Mirko Paiardini. Baricitinib treatment resolves lower-airway macrophage inflammation and neutrophil recruitment in SARS-CoV-2-infected rhesus macaques. *Cell*, 184(2):460–475.e21, January 2021.

[21] Jose Luis Rodriguez-Garcia, Gines Sanchez-Nievas, Juan Arevalo-Serrano, Cristina Garcia-Gomez, Jose Maria Jimenez-Vizuete, and Elisa Martinez-Alfaro. Baricitinib improves respiratory function in patients treated with corticosteroids for SARS-CoV-2 pneumonia: an observational cohort study. *Rheumatology*, 60(1):399–407, 10 2020.

[22] Ennio G. Favalli, Martina Biggioggero, Gabriella Maioli, and Roberto Caporali. Baricitinib for COVID-19: a suitable treatment? *The Lancet Infectious Diseases*, 20(9):1012–1013, September 2020.

[23] Vincenzo Bronte, Stefano Ugel, Elisa Tinazzi, Antonio Vella, Francesco De Sanctis, Stefania Canè, Veronica Batani, Rosalinda Trovato, Alessandra Fiore, Varvara Petrova, Francesca Hofer, Roza Maria Barouni, Chiara Musiu, Simone Caligola, Laura Pinton, Lorena Torroni, Enrico Polati, Katia Donadello, Simonetta Friso, Francesca Pizzolo, Manuela Iezzi, Federica Facciotti, Pier Giuseppe Pelicci, Daniela Righetti, Paolo Bazzoni, Mariaelisa Rampudda, Andrea Comel, Walter Mosaner, Claudio Lunardi, and Oliviero Olivieri. Baricitinib restrains the immune dysregulation in patients with severe COVID-19. *The Journal of Clinical Investigation*, 130(12):6409–6416, 12 2020.

[24] Alexey Stukalov, Virginie Girault, Vincent Grass, Ozge Karayel, Valter Bergant, Christian Urban, Darya A. Haas, Yiqi Huang, Lila Oubraham, Anqi Wang, M. Sabri Hamad, Antonio Piras, Fynn M. Hansen, Maria C. Tanzer, Igor Paron, Luca Zinzula, Thomas Engleitner, Maria Reinecke, Teresa M. Lavacca, Rosina Ehmann, Roman Wölfel, Jörg Jores, Bernhard Kuster, Ulrike Protzer,

Roland Rad, John Ziebuhr, Volker Thiel, Pietro Scaturro, Matthias Mann, and Andreas Pichlmair. Multilevel proteomics reveals host perturbations by SARS-CoV-2 and sars-cov. *Nature*, 2021.

[25] Troy G. Seelhammer, Daniel Plack, Amos Lal, and Christoph G. S. Nabzdyk. COVID-19 and ecmo: An unhappy marriage of endothelial dysfunction and hemostatic derangements. *Journal of Cardiothoracic and Vascular Anesthesia*, 34(12):3193–3196, December 2020.

[26] Troy G. Seelhammer, Phillip Rowse, and Suraj Yalamuri. Bivalirudin for maintenance anticoagulation during venovenous extracorporeal membrane oxygenation for COVID-19. *Journal of cardiothoracic and vascular anesthesia*, 35(32660924):1149–1153, April 2021.

[27] Sara Galimberti, Chiara Baldini, Claudia Baratè, Federica Ricci, Serena Balducci, Susanna Grassi, Francesco Ferro, Gabriele Buda, Edoardo Benedetti, Rita Fazzi, Laura Baglietto, Ersilia Lucenteforte, Antonello Di Paolo, and Mario Petrini. The cov-2 outbreak: how hematologists could help to fight covid-19. *Pharmacological Research*, 157:104866, 2020.

[28] Carmen Mirabelli, Jesse W. Wotring, Charles J. Zhang, Sean M. McCarty, Reid Fursmidt, Tristan Frum, Namrata S. Kadambi, Anya T. Amin, Teresa R. O'Meara, Carla D. Pretto, Jason R. Spence, Jessie Huang, Konstantinos D. Alysandratos, Darrell N. Kotton, Samuel K. Handelman, Christiane E. Wobus, Kevin J. Weatherwax, George A. Mashour, Matthew J. O'Meara, and Jonathan Z. Sexton. Morphological cell profiling of SARS-CoV-2 infection identifies drug repurposing candidates for COVID-19. *bioRxiv*, 2020.

[29] Li Yang, Rong-juan Pei, Heng Li, Xin-na Ma, Yu Zhou, Feng-hua Zhu, Pei-lan He, Wei Tang, Yecheng Zhang, Jin Xiong, Shu-qi Xiao, Xian-kun Tong, Bo Zhang, and Jian-ping Zuo. Identification of SARS-CoV-2 entry inhibitors among already approved drugs. *Acta Pharmacologica Sinica*, 2020.

[30] Pradipta R. Ray, Andi Wangzhou, Nizar Ghneim, Muhammad S. Yousuf, Candler Paige, Diana Tavares-Ferreira, Juliet M. Mwirigi, Stephanie Shiers, Ishwarya Sankaranarayanan, Amelia J. McFarland, Sanjay V. Neerukonda, Steve Davidson, Gregory Dussor, Michael D. Burton, and Theodore J. Price. A pharmacological interactome between COVID-19 patient samples and human sensory neurons reveals potential drivers of neurogenic pulmonary dysfunction. *Brain, behavior, and immunity*, 89(32497778):559–568, October 2020.

[31] José Adão Carvalho Nascimento Junior, Anamaria Mendonça Santos, Lucindo José Quintans-Júnior, Cristiani Isabel Banderó Walker, Lysandro Pinto Borges, and Mairim Russo Serafini. Sars, mers and SARS-CoV-2 (COVID-19) treatment: a patent review. *Expert Opinion on Therapeutic Patents*, 30(8):567–579, 2020. PMID: 32429703.

[32] Saeedeh Mohammadi, Mohammad Heidarizadeh, Mehrnaz Entesari, Ayoub Esmailpour, Mohammad Esmailpour, Rasoul Moradi, Nader Sakhaee, and Esmail Doustkhah. In silico investigation on the inhibiting role of nicotine/caffeine by blocking the s protein of SARS-CoV-2 versus ace2 receptor. *Microorganisms*, 8(10), 2020.

[33] Amin O. Elzupir. Caffeine and caffeine-containing pharmaceuticals as promising inhibitors for 3-chymotrypsin-like protease of SARS-CoV-2. *Journal of Biomolecular Structure and Dynamics*, 0(0):1–8, 2020. PMID: 33094705.

[34] Ben M. Flude, Giulio Nannetti, Paige Mitchell, Nina Compton, Chloe Richards, Meike Heurich, Andrea Brancale, Salvatore Ferla, and Marcella Bassetto. Targeting the complement serine protease masp-2 as a therapeutic strategy for coronavirus infections. *Viruses*, 13(2), 2021.

[35] Abhinit Kumar, Saurabh Loharch, Sunil Kumar, Rajesh P. Ringe, and Raman Parkesh. Exploiting cheminformatic and machine learning to navigate the available chemical space of potential small molecule inhibitors of SARS-CoV-2. *Computational and Structural Biotechnology Journal*, 19:424–438, 2021.

[36] Chiagozie O. Pickens, Catherine A. Gao, Michael Cuttica, Sean B. Smith, Lorenzo Pesce, Rogan Grant, Mengjia Kang, Luisa Morales-Nebreda, Avni A. Bavishi, Jason Arnold, Anna Pawlowski, Chao Qi, GR Scott Budinger, Benjamin D. Singer, and Richard G. Wunderink. Bacterial superinfection pneumonia in SARS-CoV-2 respiratory failure. *medRxiv*, 2021.

[37] Ole H Petersen, Oleg V Gerasimenko, and Julia V Gerasimenko. Endocytic uptake of SARS-CoV-2: the critical roles of pH, Ca2+, and NAADP. *Function*, 1(1), 06 2020. zqaa003.

[38] Canrong Wu, Yang Liu, Yueying Yang, Peng Zhang, Wu Zhong, Yali Wang, Qiqi Wang, Yang Xu, Mingxue Li, Xingzhou Li, Mengzhu Zheng, Lixia Chen, and Hua Li. Analysis of therapeutic targets for SARS-CoV-2 and discovery of potential drugs by computational methods. *Acta Pharmaceutica Sinica B*, 10(5):766–788, 2020.

[39] Phaedra Eleftheriou, Dionysia Amanatidou, Anthi Petrou, and Athina Geronikaki. In silico evaluation of the effectivity of approved protease inhibitors against the main protease of the novel SARS-CoV-2 virus. *Molecules*, 25(11), 2020.

[40] Sohini Chakraborti, Sneha Bheemireddy, and Narayanaswamy Srinivasan. Repurposing drugs against the main protease of SARS-CoV-2: mechanism-based insights supported by available laboratory and clinical data. *Mol. Omics*, 16:474–491, 2020.

[41] Weiwei Wan, Shenglin Zhu, Shufen Li, Weijuan Shang, Ruxue Zhang, Hao Li, Wei Liu, Gengfu Xiao, Ke Peng, and Leike Zhang. High-throughput screening of an fda-approved drug library identifies inhibitors against arenaviruses and SARS-CoV-2. *ACS Infect. Dis.*, November 2020.

[42] Beril Tutuncuoglu, Merve Cakir, Jyoti Batra, Mehdi Bouhaddou, Manon Eckhardt, David E. Gordon, and Nevan J. Krogan. The landscape of human cancer proteins targeted by SARS-CoV-2. *Cancer Discovery*, 10(7):916–921, 2020.

[43] Daria Sicari, Aristotelis Chatziioannou, Theodoros Koutsandreas, Roberto Sitia, and Eric Chevet. Role of the early secretory pathway in SARS-CoV-2 infection. *Journal of Cell Biology*, 219(9), 07 2020. e202006005.

[44] Mark Dittmar, Jae Seung Lee, Kanupriya Whig, Elisha Segrist, Minghua Li, Brinda Kamalia, Lauren Castellana, Kasirajan Ayyanathan, Fabian L. Cardenas-Diaz, Edward E. Morrisey, Rachel Truitt, Wenli Yang, Kellie Jurado, Kirandeep Samby, Holly Ramage, David C. Schultz, and Sara Cherry. Drug repurposing screens reveal cell-type-specific entry pathways and fda-approved drugs active against sars-cov-2. *Cell Reports*, 35(108959), April 2021.

[45] Andreia Palmeira, Emília Sousa, Aylin Köseler, Ramazan Sabirli, Tarık Gören, İbrahim Türkçüer, Özgür Kurt, Madalena M. Pinto, and M. Helena Vasconcelos. Preliminary virtual screening studies to identify grp78 inhibitors which may interfere with SARS-CoV-2 infection. *Pharmaceuticals*, 13(6), 2020.

[46] Qizhang Li, Zhiying Wang, Qiang Zheng, and Sen Liu. Potential clinical drugs as covalent inhibitors of the priming proteases of the spike protein of SARS-CoV-2. *Computational and Structural Biotechnology Journal*, 18:2200–2208, 2020.

[47] Sean Ekins, Melina Mottin, Paulo R. P. S. Ramos, Bruna K. P. Sousa, Bruno Junior Neves, Daniel H. Foil, Kimberley M. Zorn, Rodolpho C. Braga, Megan Coffee, Christopher Southan, Ana C. Puhl, and Carolina Horta Andrade. Déjà vu: Stimulating open drug discovery for SARS-CoV-2. *Drug discovery today*, 25(32320852):928–941, May 2020.

[48] Elisabetta Abruzzese, Luigiana Luciano, Francesco D'Agostino, Malgorzata Monika Trawinska, Fabrizio Pane, and Paolo De Fabritiis. SARS-CoV-2 (COVID-19) and chronic myeloid leukemia (CML): a case report and review of abl kinase involvement in viral infection): a case report and review of ABL kinase involvement in viral infection. *Mediterranean journal of hematology and infectious diseases*, 12(32395220):e2020031–e2020031, May 2020.

[49] Zhen Qiao, Hongtao Zhang, Hai-Feng Ji, and Qian Chen. Computational view toward the inhibition of SARS-CoV-2 spike glycoprotein and the 3cl protease. *Computation*, 8(2), 2020.

[50] Ivonne Buitrón-González, Giovanny Aguilera-Durán, and Antonio Romo-Mancillas. In-silico drug repurposing study: Amprenavir, enalaprilat, and plerixafor, potential drugs for destabilizing the SARS-CoV-2 s-protein-angiotensin-converting enzyme 2 complex. *Results in Chemistry*, 3:100094, 2021.

[51] Tamizhini Loganathan, Srimathy Ramachandran, Prakash Shankaran, Devipriya Nagarajan, and Suma Mohan S. Host transcriptome-guided drug repurposing for COVID-19 treatment: a meta-analysis based approach. *PeerJ*, 8(32566414):e9357–e9357, June 2020.

[52] Mahmoud Kandeel, Alaa H. M. Abdelrahman, Kentaro Oh-Hashi, Abdelazim Ibrahim, Katharigatta N. Venugopala, Mohamed A. Morsy, and Mahmoud A. A. Ibrahim. Repurposing of fda-approved antivirals, antibiotics, anthelmintics, antioxidants, and cell protectives against SARS-CoV-2 papain-like protease. *Journal of Biomolecular Structure and Dynamics*, 0(0):1–8, 2020. PMID: 32597315.

[53] Naveen Narayanan and Deepak T. Nair. Ritonavir may inhibit exoribonuclease activity of nsp14 from the SARS-CoV-2 virus and potentiate the activity of chain terminating drugs. *International Journal of Biological Macromolecules*, 168:272–278, 2021.

[54] Kaleb B. Tsegay, Christiana M. Adeyemi, Edward P. Gniffke, D. Noah Sather, John K. Walker, and Stephen E. P. Smith. A repurposed drug screen identifies compounds that inhibit the binding of the COVID-19 spike protein to ace2. *bioRxiv*, 2021.

[55] M. H. Raymonda, J. H. Ciesla, M. Monaghan, J. Leach, G. Asantewaa, L.A. Smorodintsev-Schiller, M. M. Lutz, X. L. Schafer, T. Takimoto, S. Dewhurst, J. Munger, and I. S. Harris. Pharmacologic profiling reveals lapatinib as a novel antiviral against SARS-CoV-2 in vitro. *bioRxiv*, 2020.

[56] Mehdi Bouhaddou, Danish Memon, Bjoern Meyer, Kris M. White, Veronica V. Rezelj, Miguel Correa Marrero, Benjamin J. Polacco, James E. Melnyk, Svenja Ulferts, Robyn M. Kaake, Jyoti Batra, Alicia L. Richards, Erica Stevenson, David E. Gordon, Ajda Rojc, Kirsten Obernier, Jacqueline M. Fabius, Margaret Soucheray, Lisa Miorin, Elena Moreno, Cassandra Koh, Quang Dinh Tran, Alexandra Hardy, Rémy Robinot, Thomas Vallet, Benjamin E. Nilsson-Payant, Claudia Hernandez-Armenta, Alistair Dunham, Sebastian Weigang, Julian Knerr, Maya Modak, Diego Quintero, Yuan Zhou, Aurelien Dugourd, Alberto Valdeolivas, Trupti Patil, Qiongyu Li, Ruth Hüttenhain, Merve Cakir, Monita Muralidharan, Minkyu Kim, Gwendolyn Jang, Beril Tutuncuoglu, Joseph Hiatt, Jeffrey Z. Guo, Jiewei Xu, Sophia Bouhaddou, Christopher J. P. Mathy, Anna Gaulton, Emma J. Manners, Eloy Félix, Ying Shi, Marisa Goff, Jean K. Lim, Timothy McBride, Michael C. O'Neal, Yiming Cai, Jason C. J. Chang, David J. Broadhurst, Saker Klippsten, Emmie De wit, Andrew R. Leach, Tanja Kortemme, Brian Shoichet, Melanie Ott, Julio Saez-Rodriguez, Benjamin R. tenOever, R. Dyche Mullins, Elizabeth R. Fischer, Georg Kochs, Robert Grosse, Adolfo García-Sastre, Marco Vignuzzi, Jeffery R. Johnson, Kevan M. Shokat, Danielle L. Swaney, Pedro Beltrao, and Nevan J. Krogan. The global phosphorylation landscape of SARS-CoV-2 infection. *Cell*, 182(3):685–712.e19, August 2020.

[57] Manoj Kumar Gupta, Sarojamma Vemula, Ravindra Donde, Gayatri Gouda, Lambodar Behera, and Ramakrishna Vadde. In-silico approaches to detect inhibitors of the human severe acute respiratory syndrome coronavirus envelope protein ion channel. *Journal of biomolecular structure and dynamics*, 39:2617–2627, Apr 2021.

[58] Selvaraj Arokiyaraj, Antony Stalin, Balakrishnan Senthamarai Kannan, and Hakdong Shin. Geranii herba as a potential inhibitor of SARS-CoV-2 main 3CLpro, spike RBD, and regulation of unfolded protein response: An in silico approach. *Antibiotics*, 9(12), 2020.

[59] Shabbir Muhammad, Sayyeda Hira Hassan, Abdullah G. Al-Sehemi, Hafiz Abdullah Shakir, Muhammad Khan, Muhammad Irfan, and Javed Iqbal. Exploring the new potential antiviral constituents of moringa oliefera for sars-cov-2 pathogenesis: An in silico molecular docking and dynamic studies. *Chemical Physics Letters*, 767:138379, 2021.

[60] Anand Kumar Pandey and Shalja Verma. An in-silico evaluation of dietary components for structural inhibition of sars-cov-2 main protease. *Journal of Biomolecular Structure and Dynamics*, 0(0):1–7, 2020. PMID: 32811367.

[61] Victoria Adeola Falade, Temitope Isaac Adelusi, Ibrahim Olaide Adedotun, Misbaudeen Abdul-Hammed, Teslim Alabi Lawal, and Saheed Alabi Agboluaje. In silico investigation of saponins and

tannins as potential inhibitors of SARS-CoV-2 main protease (m(pro)). *In silico pharmacology*, 9:9, 2021.

[62] Tin-Yun Ho, Shih-Lu Wu, Jaw-Chyun Chen, Chia-Cheng Li, and Chien-Yun Hsiang. Emodin blocks the sars coronavirus spike protein and angiotensin-converting enzyme 2 interaction. *Antiviral Research*, 74(2):92–101, 2007.

[63] Rohit Shetty, Arkasubhra Ghosh, Santosh G. Honavar, Pooja Khamar, and Swaminathan Sethu. Therapeutic opportunities to manage COVID-19/SARS-CoV-2 infection: Present and future. *Indian Journal of Ophthalmology*, 68(5), 2020.

[64] Dwight L. McKee, Ariane Sternberg, Ulrike Stange, Stefan Laufer, and Cord Naujokat. Candidate drugs against SARS-CoV-2 and COVID-19. *Pharmacological Research*, 157:104859, 2020.

[65] Ehsaneh Khodadadi, Parham Maroufi, Ehsan Khodadadi, Isabella Esposito, Khudaverdi Ganbarov, Silvano Espsoito, Mehdi Yousefi, Elham Zeinalzadeh, and Hossein Samadi Kafil. Study of combining virtual screening and antiviral treatments of the sars-cov-2 (covid-19). *Microbial Pathogenesis*, 146:104241, 2020.

[66] Faezeh Sadat Hosseini and Massoud Amanlou. Anti-hcv and anti-malaria agent, potential candidates to repurpose for coronavirus infection: Virtual screening, molecular docking, and molecular dynamics simulation study. *Life Sciences*, 258:118205, 2020.

[67] Yuanyuan Jiang, Lanxin Liu, Morenci Manning, Madison Bonahoom, Aaron Lotvola, Zhe Yang, and Zeng-Quan Yang. Structural analysis, virtual screening and molecular simulation to identify potential inhibitors targeting 2'-o-ribose methyltransferase of SARS-CoV-2 coronavirus. *Journal of Biomolecular Structure and Dynamics*, 0(0):1–16, 2020. PMID: 33016237.

[68] Uma Shankar, Neha Jain, Prativa Majee, Subodh Kumar Mishra, Brijesh Rathi, and Amit Kumar. Potential Drugs Targeting Nsp16 Protein May Corroborates a Promising Approach to Combat SARSCoV-2 Virus, 5 2020.

[69] Nouredine Behloul, Sarra Baha, Yuqian Guo, Zhifang Yang, Ruihua Shi, and Jihong Meng. In silico identification of strong binders of the SARS-CoV-2 receptor-binding domain. *European Journal of Pharmacology*, 890:173701, 2021.

[70] Mohammad Sadeq Mottaqi, Fatemeh Mohammadipanah, and Hedieh Sajedi. Contribution of machine learning approaches in response to SARS-CoV-2 infection. *Informatics in medicine unlocked*, 23:100526, 2021.

[71] Bahareh Rasaeifar, Patricia Gomez-Gutierrez, and Juan J. Perez. Molecular features of non-selective small molecule antagonists of the bradykinin receptors. *Pharmaceuticals*, 13(9), 2020.

[72] Nihad A. M. Al-Rashedi, Murad G. Munahi, and Laith AH ALObaidi. Prediction of potential inhibitors against SARS-CoV-2 endoribonuclease: RNA immunity sensing. *Journal of Biomolecular Structure and Dynamics*, 0(0):1–14, 2020. PMID: 33357040.

[73] Kedar Sharma, Sudhir Morla, Arun Goyal, and Sachin Kumar. Computational guided drug repurposing for targeting 2'-o-ribose methyltransferase of SARS-CoV-2. *Life sciences*, 259(32738360):118169–118169, October 2020.

[74] Poonam Kalhotra, Veera C. S. R. Chittepu, Guillermo Osorio-Revilla, and Tzayhri Gallardo-Velazquez. Field-template, qsar, ensemble molecular docking, and 3d-rism solvation studies expose potential of fda-approved marine drugs as sars-covid-2 main protease inhibitors. *Molecules*, 26(4), 2021.

[75] Mitsuhiro Machitani, Mami Yasukawa, Jotaro Nakashima, Yasuhiro Furuichi, and Kenkichi Masutomi. Rna-dependent rna polymerase, rdrp, a promising therapeutic target for cancer and potentially COVID-19. *Cancer Science*, 111(11):3976–3984, 2020.

[76] Pei-Gang Wang, Dong-Jiang Tang, Zhan Hua, Zai Wang, and Jing An. Sunitinib reduces the infection of sars-cov, mers-cov and SARS-CoV-2 partially by inhibiting ap2m1 phosphorylation. *Cell Discovery*, 6(1):71, 2020.

[77] Michele Catanzaro, Francesca Fagiani, Marco Racchi, Emanuela Corsini, Stefano Govoni, and Cristina Lanni. Immune response in COVID-19: addressing a pharmacological challenge by targeting pathways triggered by SARS-CoV-2. *Signal Transduction and Targeted Therapy*, 5(1):84, 2020.

[78] Sepideh Sadegh, Julian Matschinske, David B. Blumenthal, Gihanna Galindez, Tim Kacprowski, Markus List, Reza Nasirigerdeh, Mhaned Oubounyt, Andreas Pichlmair, Tim Daniel Rose, Marisol Salgado-Albarrán, Julian Späth, Alexey Stukalov, Nina K. Wenke, Kevin Yuan, Josch K. Pauling, and Jan Baumbach. Exploring the SARS-CoV-2 virus-host-drug interactome for drug repurposing. *Nature Communications*, 11(1):3518, 2020.

[79] Ana Cristina Breithaupt-Faloppa, Cristiano de Jesus Correia, Carla Máximo Prado, Roberta Sessa Stilhano, Rodrigo Portes Ureshino, and Luiz Felipe Pinho Moreira. 17β-estradiol, a potential ally to alleviate SARS-CoV-2 infection. *Clinics (Sao Paulo, Brazil)*, 75(32490931):e1980–e1980, 2020.

[80] Gennadi V. Glinsky. Tripartite combination of candidate pandemic mitigation agents: Vitamin d, quercetin, and estradiol manifest properties of medicinal agents for targeted mitigation of the COVID-19 pandemic defined by genomics-guided tracing of SARS-CoV-2 targets in human cells. *Biomedicines*, 8(5), 2020.

[81] Ute Seeland, Flaminia Coluzzi, Maurizio Simmaco, Cameron Mura, Philip E. Bourne, Max Heiland, Robert Preissner, and Saskia Preissner. Evidence for treatment with estradiol for women with SARS-CoV-2 infection. *BMC Medicine*, 18(1):369, 2020.

[82] Sandro La Vignera, Rossella Cannarella, Rosita A. Condorelli, Francesco Torre, Antonio Aversa, and Aldo E. Calogero. Sex-specific SARS-CoV-2 mortality: Among hormone-modulated ace2 expression, risk of venous thromboembolism and hypovitaminosis d. *International Journal of Molecular Sciences*, 21(8), 2020.

[83] Soodeh Mahdian, Mahboobeh Zarrabi, Yunes Panahi, and Somayyeh Dabbagh. Repurposing fda-approved drugs to fight COVID-19 using in silico methods: Targeting SARS-CoV-2 rdrp enzyme and host cell receptors (ace2, cd147) through virtual screening and molecular dynamic simulations. *Informatics in Medicine Unlocked*, 23:100541, 2021.

[84] Chan Yang, Xiaoyan Pan, Yuan Huang, Chen Cheng, Xinfeng Xu, Yan Wu, Yunxia Xu, Weijuan Shang, Xiaoge Niu, Yihong Wan, Zhaofeng Li, Rong Zhang, Shuwen Liu, Gengfu Xiao, and Wei Xu. Drug repurposing of itraconazole and estradiol benzoate against COVID-19 by blocking SARS-CoV-2 spike protein-mediated membrane fusion. *Adv. Therap.*, n/a(n/a):2000224, February 2021.

[85] Anna C. Aschenbrenner, Maria Mouktaroudi, Benjamin Krämer, Marie Oestreich, Nikolaos Antonakos, Melanie Nuesch-Germano, Konstantina Gkizeli, Lorenzo Bonaguro, Nico Reusch, Kevin Baßler, Maria Saridaki, Rainer Knoll, Tal Pecht, Theodore S. Kapellos, Sarandia Doulou, Charlotte Kröger, Miriam Herbert, Lisa Holsten, Arik Horne, Ioanna D. Gemünd, Nikoletta Rovina, Shobhit Agrawal, Kilian Dahm, Martina van Uelft, Anna Drews, Lena Lenkeit, Niklas Bruse, Jelle Gerretsen, Jannik Gierlich, Matthias Becker, Kristian Händler, Michael Kraut, Heidi Theis, Simachew Mengiste, Elena De Domenico, Jonas Schulte-Schrepping, Lea Seep, Jan Raabe, Christoph Hoffmeister, Michael ToVinh, Verena Keitel, Gereon Rieke, Valentina Talevi, Dirk Skowasch, N. Ahmad Aziz, Peter Pickkers, Frank L. van de Veerdonk, Mihai G. Netea, Joachim L. Schultze, Matthijs Kox, Monique M. B. Breteler, Jacob Nattermann, Antonia Koutsoukou, Evangelos J. Giamarellos-Bourboulis, Thomas Ulas, Janine Altmüller, Angel Angelov, Robert Bals, Alexander Bartholomäus, Anke Becker, Michael Bitzer, Ezio Bonifacio, Peer Bork, Nicolas Casadei, Thomas Clavel, Maria Colome-Tatche, Andreas Diefenbach, Alexander Dilthey, Nicole Fischer, Konrad Förstner, Sören Franzenburg, Julia-Stefanie Frick, Gisela Gabernet, Julien Gagneur, Tina Ganzenmüller, Siri Göpel, Alexander Goesmann, Torsten Hain, André Heimbach, Michael Hummel, Angelika Iftner, Thomas Iftner, Stefan Janssen, Jörn Kalinowski, René Kallies, Birte Kehr, Andreas

Keller, Sarah Kim-Hellmuth, Christoph Klein, Oliver Kohlbacher, Karl Köhrer, Jan Korbel, Denise Kühnert, Ingo Kurth, Markus Landthaler, Yang Li, Kerstin Ludwig, Oliwia Makarewicz, Manja Marz, Alice McHardy, Christian Mertes, Markus Nöthen, Peter Nürnberg, Uwe Ohler, Stephan Ossowski, Jörg Overmann, Klaus Pfeffer, Anna R. Poetsch, Alfred Pühler, Nikolaus Rajewsky, Markus Ralser, Olaf Rieß, Stephan Ripke, Ulisses Nunes da Rocha, Philip Rosenstiel, Antoine-Emmanuel Saliba, Leif Erik Sander, Birgit Sawitzki, Philipp Schiffer, Wulf Schneider, Eva-Christina Schulte, Joachim L. Schultze, Alexander Sczyrba, Yogesh Singh, Michael Sonnabend, Oliver Stegle, Jens Stoye, Fabian Theis, Janne Vehreschild, Jörg Vogel, Max von Kleist, Andreas Walker, Jörn Walter, Dagmar Wieczorek, Sylke Winkler, John Ziebuhr, and German C. O. V. I. D.-19 Omics Initiative (DeCOI). Disease severity-specific neutrophil signatures in blood transcriptomes stratify COVID-19 patients. *Genome Medicine*, 13(1):7, 2021.

[86] Gennaro Ciliberto, Rita Mancini, and Marco G. Paggi. Drug repurposing against COVID-19: focus on anticancer agents. *Journal of Experimental & Clinical Cancer Research*, 39(1):86, 2020.

[87] Antonio Romanelli and Silvia Mascolo. Sirolimus to treat SARS-CoV-2 infection: an old drug for a new disease. *J Res Clin Med*, 8(1):44–44, 2020.

[88] Evelyne Bischof, Richard C. Siow, Alex Zhavoronkov, and Matt Kaeberlein. The potential of rapalogs to enhance resilience against SARS-CoV-2 infection and reduce the severity of COVID-19. *The Lancet Healthy Longevity*, 2(2):e105–e111, February 2021.

[89] Farshad Abedi, Ramin Rezaee, and Gholamreza Karimi. Plausibility of therapeutic effects of rho kinase inhibitors against severe acute respiratory syndrome coronavirus 2 (COVID-19). *Pharmacological Research*, 156:104808, 2020.

[90] Soudeh Ghafouri-Fard, Rezvan Noroozi, Mir Davood Omrani, Wojciech Branicki, Ewelina Pośpiech, Arezou Sayad, Krzysztof Pyrc, Paweł P. Łabaj, Reza Vafaee, Mohammad Taheri, and Marek Sanak. Angiotensin converting enzyme: A review on expression profile and its association with human disorders with special focus on SARS-CoV-2 infection. *Vascular Pharmacology*, 130:106680, 2020.

[91] Matija Fenrich, Stefan Mrdenovic, Marta Balog, Svetlana Tomic, Milorad Zjalic, Alen Roncevic, Dario Mandic, Zeljko Debeljak, and Marija Heffer. SARS-CoV-2 dissemination through peripheral nerves explains multiple organ injury. *Frontiers in Cellular Neuroscience*, 14:229, 2020.

[92] Ahmed S. Gouda and Bruno Mégarbane. Snake venom-derived bradykinin-potentiating peptides: A promising therapy for COVID-19? *Drug Development Research*, 82(1):38–48, 2021.

[93] Rahul Parit and Sridhar Jayavel. Association of ace inhibitors and angiotensin type ii blockers with ace2 overexpression in COVID-19 comorbidities: A pathway-based analytical study. *European Journal of Pharmacology*, 896:173899, 2021.

[94] Dandan Wu and Xuexian O. Yang. Th17 responses in cytokine storm of COVID-19: An emerging target of jak2 inhibitor fedratinib. *Journal of Microbiology, Immunology and Infection*, 53(3):368–370, 2020.

[95] Ahmad Abu Turab Naqvi, Kisa Fatima, Taj Mohammad, Urooj Fatima, Indrakant K. Singh, Archana Singh, Shaikh Muhammad Atif, Gururao Hariprasad, Gulam Mustafa Hasan, and Md. Imtaiyaz Hassan. Insights into SARS-CoV-2 genome, structure, evolution, pathogenesis and therapies: Structural genomics approach. *Biochimica et Biophysica Acta (BBA) - Molecular Basis of Disease*, 1866(10):165878, 2020.

[96] Lucia Lisi, Pedro Miguel Lacal, Maria Luisa Barbaccia, and Grazia Graziani. Approaching coronavirus disease 2019: Mechanisms of action of repurposed drugs with potential activity against SARS-CoV-2. *Biochemical Pharmacology*, 180:114169, 2020.

[97] Mizuki Yamamoto, Maki Kiso, Yuko Sakai-Tagawa, Kiyoko Iwatsuki-Horimoto, Masaki Imai, Makoto Takeda, Noriko Kinoshita, Norio Ohmagari, Jin Gohda, Kentaro Semba, Zene Matsuda, Yasushi Kawaguchi, Yoshihiro Kawaoka, and Jun-ichiro Inoue. The anticoagulant nafamostat potently inhibits SARS-CoV-2 s protein-mediated fusion in a cell fusion assay system and viral infection in vitro in a cell-type-dependent manner. *Viruses*, 12(6), 2020.

[98] Markus Hoffmann, Simon Schroeder, Hannah Kleine-Weber, Marcel A. Müller, Christian Drosten, and Stefan Pöhlmann. Nafamostat mesylate blocks activation of SARS-CoV-2: New treatment option for COVID-19. *Antimicrobial Agents and Chemotherapy*, 64(6), 2020.

[99] Hubert Hondermarck, Nathan W. Bartlett, and Victor Nurcombe. The role of growth factor receptors in viral infections: An opportunity for drug repurposing against emerging viral diseases such as COVID-19? *FASEB BioAdvances*, 2(5):296–303, 2020.

[100] Siddhi Joshi, Maithili Joshi, and Mariam S Degani. Tackling SARS-CoV-2: proposed targets and repurposed drugs. *Future Medicinal Chemistry*, 12(17):1579–1601, 2020. PMID: 32564623.

[101] Balaji Gowrivel Vijayakumar, Deepthi Ramesh, Annu Joji, Jayadharini Jayachandra prakasan, and Tharanikkarasu Kannan. In silico pharmacokinetic and molecular docking studies of natural flavonoids and synthetic indole chalcones against essential proteins of SARS-CoV-2. *European Journal of Pharmacology*, 886:173448, 2020.

[102] C. M. Clemente, M. I. Freiberger, S. Ravetti, D. M. Beltramo, and A. G. Garro. An in silico analysis of ibuprofen enantiomers in high concentrations of sodium chloride with SARS-CoV-2 main protease. *Journal of Biomolecular Structure and Dynamics*, 0(0):1–12, 2021. PMID: 33459192.

[103] Jennifer S. Chen, Mia Madel Alfajaro, Ryan D. Chow, Jin Wei, Renata B. Filler, Stephanie C. Eisenbarth, and Craig B. Wilen. Nonsteroidal anti-inflammatory drugs dampen the cytokine and antibody response to SARS-CoV-2 infection. *Journal of Virology*, 95(7), 2021.

[104] Helong Zhao, Michelle Mendenhall, and Michael W. Deininger. Imatinib is not a potent anti-SARS-CoV-2 drug. *Leukemia*, 34(11):3085–3087, 2020.

[105] David Bernal-Bello, Beatriz Jaenes-Barrios, Alejandro Morales-Ortega, José Manuel Ruiz-Giardin, Virginia García-Bermúdez, Begoña Frutos-Pérez, Ana Isabel Farfán-Sedano, Cristina de Ancos-Aracil, Fernando Bermejo, Mario García-Gil, Antonio Zapatero-Gaviria, and Juan Víctor San Martín-López. Imatinib might constitute a treatment option for lung involvement in COVID-19. *Autoimmunity Reviews*, 19(7):102565, 2020. Special issue COVID19 and Autoimmunity.

[106] Allan Sauvat, Fabiola Ciccosanti, Francesca Colavita, Martina Di Rienzo, Concetta Castilletti, Maria Rosaria Capobianchi, Oliver Kepp, Laurence Zitvogel, Gian Maria Fimia, Mauro Piacentini, and Guido Kroemer. On-target versus off-target effects of drugs inhibiting the replication of SARS-CoV-2. *Cell Death & Disease*, 11(8):656, 2020.

[107] Yuling Han, Xiaohua Duan, Liuliu Yang, Benjamin E. Nilsson-Payant, Pengfei Wang, Fuyu Duan, Xuming Tang, Tomer M. Yaron, Tuo Zhang, Skyler Uhl, Yaron Bram, Chanel Richardson, Jiajun Zhu, Zeping Zhao, David Redmond, Sean Houghton, Duc-Huy T. Nguyen, Dong Xu, Xing Wang, Jose Jessurun, Alain Borczuk, Yaoxing Huang, Jared L. Johnson, Yuru Liu, Jenny Xiang, Hui Wang, Lewis C. Cantley, Benjamin R. tenOever, David D. Ho, Fong Cheng Pan, Todd Evans, Huanhuan Joyce Chen, Robert E. Schwartz, and Shuibing Chen. Identification of SARS-CoV-2 inhibitors using lung and colonic organoids. *Nature*, 589(7841):270–275, 2021.

[108] Vaibhav Tiwari, Jacob C. Beer, Nehru Viji Sankaranarayanan, Michelle Swanson-Mungerson, and Umesh R. Desai. Discovering small-molecule therapeutics against SARS-CoV-2. *Drug Discovery Today*, 25(8):1535–1544, 2020.

[109] Jennifer Santos, Stephanie Brierley, Mohit J. Gandhi, Michael A. Cohen, Phillip C. Moschella, and Arwen B. L. Declan. Repurposing therapeutics for potential treatment of SARS-CoV-2: A review. *Viruses*, 12(7), 2020.

[110] Stuart Weston, Christopher M. Coleman, Robert Haupt, James Logue, Krystal Matthews, Yize Li, Hanako M. Reyes, Susan R. Weiss, and Matthew B. Frieman. Broad anti-coronavirus activity of food and drug administration-approved drugs against SARS-CoV-2 in vitro and sars-cov in vivo. *Journal of Virology*, 94(21), 2020.

[111] Catherine Z. Chen, Miao Xu, Manisha Pradhan, Kirill Gorshkov, Jennifer D. Petersen, Marco R. Straus, Wei Zhu, Paul Shinn, Hui Guo, Min Shen, Carleen Klumpp-Thomas, Samuel G. Michael, Joshua Zimmerberg, Wei Zheng, and Gary R. Whittaker. Identifying SARS-CoV-2 entry inhibitors through drug repurposing screens of sars-s and mers-s pseudotyped particles. *ACS Pharmacol. Transl. Sci.*, 3(6):1165–1175, December 2020.

[112] Giulia Culletta, Maria Rita Gulotta, Ugo Perricone, Maria Zappalà, Anna Maria Almerico, and Marco Tutone. Exploring the SARS-CoV-2 proteome in the search of potential inhibitors via structure-based pharmacophore modeling/docking approach. *Computation*, 8(3), 2020.

[113] K Baby, S Maity, CH Mehta, A Suresh, UY Nayak, and Y Nayak. Targeting SARS-CoV-2 RNA-dependent RNA polymerase: An in silico drug repurposing for COVID-19 [version 1; peer review: 2 approved]. *F1000Research*, 9(1166), 2020.

[114] Alisa Pavel, Giusy del Giudice, Antonio Federico, Antonio Di Lieto, Pia A S Kinaret, Angela Serra, and Dario Greco. Integrated network analysis reveals new genes suggesting COVID-19 chronic effects and treatment. *Briefings in Bioinformatics*, 22(2):1430–1441, 02 2021.

[115] Sirle Saul, Marwah Karim, Pei Tzu Huang, Luca Ghita, Winston Chiu, Sathish Kumar, Nishank Bhalla, Pieter Leyssen, Courtney Cohen, Kathleen Huie, Courtney Tindle, Malaya Sahoo, Mamdouh Sibai, Benjamin A. Pinsky, Soumita Das, Pradipta Ghosh, John Dye, David Solow-Cordero, Jing Jin, Dirk Jochmans, Johan Neyts, Aarthi Narayanan, Steven De Jonghe, and Shirit Einav. Pan-erbb inhibition protects from SARS-CoV-2 replication, inflammation, and injury. *bioRxiv*, 2021.

[116] Hira Maab, Faryal Mustafa, and Shajeea Arshad Ali. Anti-inflammatory aspects of lidocaine: a neglected therapeutic stance for COVID-19. *Heart & lung : the journal of critical care*, 49(32980171):877–878, 2020.

[117] Ziad A. Ali and Rif S. El-Mallakh. Nebulized lidocaine in COVID-19, an hypothesis. *Medical Hypotheses*, 144:109947, 2020.

[118] Seyedeh Zahra Mousavi, Mojdeh Rahmanian, and Ashkan Sami. A connectivity map-based drug repurposing study and integrative analysis of transcriptomic profiling of SARS-CoV-2 infection. *Infection, Genetics and Evolution*, 86:104610, 2020.

[119] Carlos Spuch, Marta López-García, Tania Rivera-Baltanás, Daniela Rodrígues-Amorím, and José M. Olivares. Does lithium deserve a place in the treatment against COVID-19? a preliminary observational study in six patients, case report. *Frontiers in Pharmacology*, 11:1347, 2020.

[120] Abdallah Barjas Qaswal, Aiman Suleiman, Hasan Guzu, Taima'a Harb, and Bashir Atiyat. The potential role of lithium as an antiviral agent against SARS-CoV-2 via membrane depolarization: Review and hypothesis. *Scientia Pharmaceutica*, 89(1), 2021.

[121] Lopamudra Dey, Sanjay Chakraborty, and Anirban Mukhopadhyay. Machine learning techniques for sequence-based prediction of viral–host interactions between SARS-CoV-2 and human proteins. *Biomedical Journal*, 43(5):438–450, 2020.

[122] Alfonso Trezza, Daniele Iovinelli, Annalisa Santucci, Filippo Prischi, and Ottavia Spiga. An integrated drug repurposing strategy for the rapid identification of potential SARS-CoV-2 viral inhibitors. *Scientific Reports*, 10(1):13866, 2020.

[123] Alicia Jiménez-Alberto, Rosa María Ribas-Aparicio, Gerardo Aparicio-Ozores, and Juan A. Castelán-Vega. Virtual screening of approved drugs as potential SARS-CoV-2 main protease inhibitors. *Computational Biology and Chemistry*, 88:107325, 2020.

[124] David E. Gordon, Gwendolyn M. Jang, Mehdi Bouhaddou, Jiewei Xu, Kirsten Obernier, Kris M. White, Matthew J. O'Meara, Veronica V. Rezelj, Jeffrey Z. Guo, Danielle L. Swaney, Tia A. Tummino, Ruth Hüttenhain, Robyn M. Kaake, Alicia L. Richards, Beril Tutuncuoglu, Helene Foussard, Jyoti Batra, Kelsey Haas, Maya Modak, Minkyu Kim, Paige Haas, Benjamin J. Polacco,

Hannes Braberg, Jacqueline M. Fabius, Manon Eckhardt, Margaret Soucheray, Melanie J. Bennett, Merve Cakir, Michael J. McGregor, Qiongyu Li, Bjoern Meyer, Ferdinand Roesch, Thomas Vallet, Alice Mac Kain, Lisa Miorin, Elena Moreno, Zun Zar Chi Naing, Yuan Zhou, Shiming Peng, Ying Shi, Ziyang Zhang, Wenqi Shen, Ilsa T. Kirby, James E. Melnyk, John S. Chorba, Kevin Lou, Shizhong A. Dai, Inigo Barrio-Hernandez, Danish Memon, Claudia Hernandez-Armenta, Jiankun Lyu, Christopher J. P. Mathy, Tina Perica, Kala Bharath Pilla, Sai J. Ganesan, Daniel J. Saltzberg, Ramachandran Rakesh, Xi Liu, Sara B. Rosenthal, Lorenzo Calviello, Srivats Venkataramanan, Jose Liboy-Lugo, Yizhu Lin, Xi-Ping Huang, YongFeng Liu, Stephanie A. Wankowicz, Markus Bohn, Maliheh Safari, Fatima S. Ugur, Cassandra Koh, Nastaran Sadat Savar, Quang Dinh Tran, Djoshkun Shengjuler, Sabrina J. Fletcher, Michael C. O'Neal, Yiming Cai, Jason C. J. Chang, David J. Broadhurst, Saker Klippsten, Phillip P. Sharp, Nicole A. Wenzell, Duygu Kuzuoglu-Ozturk, Hao-Yuan Wang, Raphael Trenker, Janet M. Young, Devin A. Cavero, Joseph Hiatt, Theodore L. Roth, Ujjwal Rathore, Advait Subramanian, Julia Noack, Mathieu Hubert, Robert M. Stroud, Alan D. Frankel, Oren S. Rosenberg, Kliment A. Verba, David A. Agard, Melanie Ott, Michael Emerman, Natalia Jura, Mark von Zastrow, Eric Verdin, Alan Ashworth, Olivier Schwartz, Christophe d'Enfert, Shaeri Mukherjee, Matt Jacobson, Harmit S. Malik, Danica G. Fujimori, Trey Ideker, Charles S. Craik, Stephen N. Floor, James S. Fraser, John D. Gross, Andrej Sali, Bryan L. Roth, Davide Ruggero, Jack Taunton, Tanja Kortemme, Pedro Beltrao, Marco Vignuzzi, Adolfo García-Sastre, Kevan M. Shokat, Brian K. Shoichet, and Nevan J. Krogan. A SARS-CoV-2 protein interaction map reveals targets for drug repurposing. *Nature*, 583(7816):459–468, 2020.

[125] Edoardo Francini, Salvatora T. Miano, Anna I. Fiaschi, and Guido Francini. Doxycycline or minocycline may be a viable treatment option against SARS-CoV-2. *Medical Hypotheses*, 144:110054, 2020.

[126] Harmanjit Singh, Ashish Kumar Kakkar, and Prerna Chauhan. Repurposing minocycline for COVID-19 management: mechanisms, opportunities, and challenges. *Expert Review of Anti-infective Therapy*, 18(10):997–1003, 2020. PMID: 32552044.

[127] Aline C. Oliveira, Elaine M. Richards, Marianthi M. Karas, Carl J. Pepine, and Mohan K. Raizada. Would repurposing minocycline alleviate neurologic manifestations of COVID-19? *Frontiers in Neuroscience*, 14:997, 2020.

[128] Giovanni Diana, Rocky Strollo, Davide Diana, Mirko Strollo, Alfredo R Galassi, and Filippo Crea. Cardiac safety and potential efficacy: two reasons for considering minocycline in place of azithromycin in COVID-19 management. *European Heart Journal - Cardiovascular Pharmacotherapy*, 05 2020. pvaa049.

[129] Shiv Bharadwaj, Kyung Eun Lee, Vivek Dhar Dwivedi, and Sang Gu Kang. Computational insights into tetracyclines as inhibitors against SARS-CoV-2 mpro via combinatorial molecular simulation calculations. *Life Sciences*, 257:118080, 2020.

[130] Kent Doi, Mahoko Ikeda, Naoki Hayase, Kyoji Moriya, Naoto Morimura, Hiromu Maehara, Shunsuke Tagami, Kazutaka Fukushima, Naho Misawa, Yutaro Inoue, Hitomi Nakamura, Daisuke Takai, Mio Kurimoto, Kurato Tokunaga, Miyuki Yamamoto, Ichiro Hirayama, Ryohei Horie, Yuri Endo, Kengo Hiwatashi, Mio Shikama, Daisuke Jubishi, Yoshiaki Kanno, Koh Okamoto, Sohei Harada, Shu Okugawa, Kohei Miyazono, Yasuyuki Seto, Jun-ichiro Inoue, and the COVID-UTH Study Group. Nafamostat mesylate treatment in combination with favipiravir for patients critically ill with covid-19: a case series. *Critical Care*, 24(1):392, 2020.

[131] Meehyun Ko, Sangeun Jeon, Wang-Shick Ryu, and Seungtaek Kim. Comparative analysis of antiviral efficacy of fda-approved drugs against SARS-CoV-2 in human lung cells. *Journal of Medical Virology*, 93(3):1403–1408, 2021.

[132] Vinicius M. Alves, Tesia Bobrowski, Cleber C. Melo-Filho, Daniel Korn, Scott Auerbach, Charles Schmitt, Eugene N. Muratov, and Alexander Tropsha. QSAR modeling of SARS-CoV Mpro inhibitors identifies sufugolix, cenicriviroc, proglumetacin, and other drugs as candidates for repurposing against SARS-CoV-2. *Molecular Informatics*, 40(1):2000113, 2021.

[133] Victor S. Stroylov and Igor V. Svitanko. Computational identification of disulfiram and neratinib as putative SARS-CoV-2 main protease inhibitors. *Mendeleev Communications*, 30(4):419–420, 2020.

[134] Valeria Cagno, Gaelle Magliocco, Caroline Tapparel, and Youssef Daali. The tyrosine kinase inhibitor nilotinib inhibits SARS-CoV-2 in vitro. *Basic & Clinical Pharmacology & Toxicology*, 128(4):621–624, 2021.

[135] Zijing Ruan, Chao Liu, Yuting Guo, Zhenqing He, Xinhe Huang, Xu Jia, and Tai Yang. SARS-CoV-2 and sars-cov: Virtual screening of potential inhibitors targeting rna-dependent rna polymerase activity (nsp12). *Journal of Medical Virology*, 93(1):389–400, 2021.

[136] Jr. Garcia, Gustavo, Arun Sharma, Arunachalam Ramaiah, Chandani Sen, Arunima Purkayastha, Donald B. Kohn, Mark S. Parcells, Sebastian Beck, Heeyoung Kim, Malina A. Bakowski, Melanie G. Kirkpatrick, Laura Riva, Karen C. Wolff, Brandon Han, Constance Yuen, David Ulmert, Prabhat K. Purbey, Phillip Scumpia, Nathan Beutler, Thomas F. Rogers, Arnab K. Chatterjee, Gülsah Gabriel, Ralf Bartenschlager, Brigitte Gomperts, Clive N. Svendsen, Ulrich A. K. Betz, Robert D. Damoiseaux, and Vaithilingaraja Arumugaswami. Antiviral drug screen identifies dna-damage response inhibitor as potent blocker of SARS-CoV-2 replication. *Cell Reports*, 35(108940), April 2021.

[137] Giuseppe Deganutti, Filippo Prischi, and Christopher A. Reynolds. Supervised molecular dynamics for exploring the druggability of the SARS-CoV-2 spike protein. *Journal of Computer-Aided Molecular Design*, 35(2):195–207, 2021.

[138] Mark Andrew White, Wei Lin, and Xiaodong Cheng. Discovery of COVID-19 inhibitors targeting the SARS-CoV-2 nsp13 helicase. *J. Phys. Chem. Lett.*, 11(21):9144–9151, November 2020.

[139] Lei Sun, Pan Li, Xiaohui Ju, Jian Rao, Wenze Huang, Lili Ren, Shaojun Zhang, Tuanlin Xiong, Kui Xu, Xiaolin Zhou, Mingli Gong, Eric Miska, Qiang Ding, Jianwei Wang, and Qiangfeng Cliff Zhang. In vivo structural characterization of the SARS-CoV-2 rna genome identifies host proteins vulnerable to repurposed drugs. *Cell*, 184(7):1865–1883.e20, 2021.

[140] Finny S. Varghese, Esther van Woudenbergh, Gijs J. Overheul, Marc J. Eleveld, Lisa Kurver, Niels van Heerbeek, Arjan van Laarhoven, Pascal Miesen, Gerco den Hartog, Marien I. de Jonge, and Ronald P. van Rij. Berberine and obatoclax inhibit sars-cov-2 replication in primary human nasal epithelial cells in vitro. *Viruses*, 13(2), 2021.

[141] Aleksandr Ianevski, Rouan Yao, Mona Høysæter Fenstad, Svetlana Biza, Eva Zusinaite, Tuuli Reisberg, Hilde Lysvand, Kirsti Løseth, Veslemøy Malm Landsem, Janne Fossum Malmring, Valentyn Oksenych, Sten Even Erlandsen, Per Arne Aas, Lars Hagen, Caroline H. Pettersen, Tanel Tenson, Jan Egil Afset, Svein Arne Nordbø, Magnar Bjørås, and Denis E. Kainov. Potential antiviral options against SARS-CoV-2 infection. *Viruses*, 12(6), 2020.

[142] Son Tung Ngo, Hung Minh Nguyen, Le Thi Thuy Huong, Pham Minh Quan, Vi Khanh Truong, Nguyen Thanh Tung, and Van V. Vu. Assessing potential inhibitors of SARS-CoV-2 main protease from available drugs using free energy perturbation simulations. *RSC Adv.*, 10:40284–40290, 2020.

[143] Malina A. Bakowski, Nathan Beutler, Emily Chen, Tu-Trinh H. Nguyen, Melanie G. Kirkpatrick, Mara Parren, Linlin Yang, James Ricketts, Anil K. Gupta, Mitchell V. Hull, Peter G. Schultz, Dennis R. Burton, Arnab K. Chatterjee, Case W. McNamara, and Thomas F. Rogers. Oral drug repositioning candidates and synergistic remdesivir combinations for the prophylaxis and treatment of COVID-19. *bioRxiv*, 2020.

[144] Maryam S. Al-Motawa, Hafsa Abbas, Patrick Wijten, Alberto de la Fuente, Mingzhan Xue, Naila Rabbani, and Paul J. Thornalley. Vulnerabilities of the SARS-CoV-2 virus to proteotoxicity—opportunity for repurposed chemotherapy of COVID-19 infection. *Frontiers in Pharmacology*, 11:1579, 2020.

[145] Mirko Cortese, Ji-Young Lee, Berati Cerikan, Christopher J. Neufeldt, Viola M.J. Oorschot, Sebastian Köhrer, Julian Hennies, Nicole L. Schieber, Paolo Ronchi, Giulia Mizzon, Inés Romero-Brey, Rachel Santarella-Mellwig, Martin Schorb, Mandy Boermel, Karel Mocaer, Marianne S. Beckwith, Rachel M. Templin, Viktoriia Gross, Constantin Pape, Christian Tischer, Jamie Frankish, Natalie K. Horvat, Vibor Laketa, Megan Stanifer, Steeve Boulant, Alessia Ruggieri, Laurent Chatel-Chaix, Yannick Schwab, and Ralf Bartenschlager. Integrative imaging reveals SARS-CoV-2-induced reshaping of subcellular morphologies. *Cell Host & Microbe*, 28(6):853–866.e5, 2020.

[146] Umesh Kalathiya, Monikaben Padariya, Marcos Mayordomo, Małgorzata Lisowska, Judith Nicholson, Ashita Singh, Maciej Baginski, Robin Fahraeus, Neil Carragher, Kathryn Ball, Juergen Haas, Alison Daniels, Ted R. Hupp, and Javier Antonio Alfaro. Highly conserved homotrimer cavity formed by the SARS-CoV-2 spike glycoprotein: A novel binding site. *Journal of Clinical Medicine*, 9(5), 2020.

[147] Rajaiah Alexpandi, Joelma Freire De Mesquita, Shunmugiah Karutha Pandian, and Arumugam Veera Ravi. Quinolines-based SARS-CoV-2 3CLpro and RdRp inhibitors and Spike-RBD-ACE2 inhibitor for drug-repurposing against COVID-19: An in silico analysis. *Frontiers in Microbiology*, 11:1796, 2020.

[148] Sebastian Günther, Patrick Y. A. Reinke, Yaiza Fernández-García, Julia Lieske, Thomas J. Lane, Helen M. Ginn, Faisal H. M. Koua, Christiane Ehrt, Wiebke Ewert, Dominik Oberthuer, Oleksandr Yefanov, Susanne Meier, Kristina Lorenzen, Boris Krichel, Janine-Denise Kopicki, Luca Gelisio, Wolfgang Brehm, Ilona Dunkel, Brandon Seychell, Henry Gieseler, Brenna Norton-Baker, Beatriz Escudero-Pérez, Martin Domaracky, Sofiane Saouane, Alexandra Tolstikova, Thomas A. White, Anna Hänle, Michael Groessler, Holger Fleckenstein, Fabian Trost, Marina Galchenkova, Yaroslav Gevorkov, Chufeng Li, Salah Awel, Ariana Peck, Miriam Barthelmess, Frank Schlünzen, P. Lourdu Xavier, Nadine Werner, Hina Andaleeb, Najeeb Ullah, Sven Falke, Vasundara Srinivasan, Bruno Alves Franca, Martin Schwinzer, Hévila Brognaro, Cromarte Rogers, Diogo Melo, Jo J. Zaitsev-Doyle, Juraj Knoska, Gisel E. Peña Murillo, Aida Rahmani Mashhour, Filip Guicking, Vincent Hennicke, Pontus Fischer, Johanna Hakanpää, Jan Meyer, Phil Gribbon, Bernhard Ellinger, Maria Kuzikov, Markus Wolf, Andrea R. Beccari, Gleb Bourenkov, David von Stetten, Guillaume Pompidor, Isabel Bento, Saravanan Panneerselvam, Ivars Karpics, Thomas R. Schneider, Maria Marta Garcia Alai, Stephan Niebling, Christian Günther, Christina Schmidt, Robin Schubert, Huijong Han, Juliane Boger, Diana C. F. Monteiro, Linlin Zhang, Xinyuanyuan Sun, Jonathan Pletzer-Zelgert, Jan Wollenhaupt, Christian G. Feiler, Manfred S. Weiss, Eike-Christian Schulz, Pedram Mehrabi, Katarina Karničar, Aleksandra Usenik, Jure Loboda, Henning Tidow, Ashwin Chari, Rolf Hilgenfeld, Charlotte Uetrecht, Russell Cox, Andrea Zaliani, Tobias Beck, Matthias Rarey, Stephan Günther, Dusan Turk, Winfried Hinrichs, Henry N. Chapman, Arwen R. Pearson, Christian Betzel, and Alke Meents. Inhibition of SARS-CoV-2 main protease by allosteric drug-binding. *bioRxiv*, 2020.

[149] Derek W. Gilroy, Roel P.H. De Maeyer, Mark Tepper, Alastair O'Brien, Mohib Uddin, Judy Chen, Daniel R. Goldstein, and Arne N. Akbar. Treating exuberant, non-resolving inflammation in the lung; implications for acute respiratory distress syndrome and COVID-19. *Pharmacology & Therapeutics*, page 107745, 2020.

[150] Zhesheng He, Wencong Zhao, Wenchao Niu, Xuejiao Gao, Xingfa Gao, Yong Gong, and Xueyun Gao. Molecules inhibit the enzyme activity of 3-chymotrypsin-like cysteine protease of SARS-CoV-2 virus: the experimental and theory studies. *bioRxiv*, 2020.

[151] Lilian Peñalver, Philipp Schmid, Dávid Szamosvári, Stefan Schildknecht, Christoph Globisch, Kevin Sawade, Christine Peter, and Thomas Böttcher. A ligand selection strategy identifies chemical probes targeting the proteases of SARS-CoV-2. *Angewandte Chemie International Edition*, 60(12):6799–6806, 2021.

[152] Amin O. Elzupir. Inhibition of SARS-CoV-2 main protease 3clpro by means of α-ketoamide and pyridone-containing pharmaceuticals using in silico molecular docking. *Journal of Molecular Structure*, 1222:128878, 2020.

[153] Irene Maffucci and Alessandro Contini. In silico drug repurposing for SARS-CoV-2 main proteinase and spike proteins. *J. Proteome Res.*, 19(11):4637–4648, November 2020.

[154] Deisy Morselli Gysi, Ítalo do Valle, Marinka Zitnik, Asher Ameli, Xiao Gan, Onur Varol, Susan Dina Ghiassian, J. J. Patten, Robert A. Davey, Joseph Loscalzo, and Albert-László Barabási. Network medicine framework for identifying drug-repurposing opportunities for COVID-19. *Proceedings of the National Academy of Sciences*, 118(19):e2025581118, 05 2021.

[155] Valeria Napolitano, Agnieszka Dabrowska, Kenji Schorpp, André Mourão, Emilia Barreto-Duran, Malgorzata Benedyk, Pawel Botwina, Stefanie Brandner, Mark Bostock, Yuliya Chykunova, Anna Czarna, Grzegorz Dubin, Tony Fröhlich, Michael Hoelscher, Malwina Jedrysik, Alex Matsuda, Katarzyna Owczarek, Magdalena Pachota, Oliver Plettenburg, Jan Potempa, Ina Rothenaigner, Florian Schlauderer, Artur Szczepanski, Kristin Greve-Isdahl Mohn, Bjorn Blomberg, Michael Sattler, Kamyar Hadian, Grzegorz Maria Popowicz, and Krzysztof Pyrc. Acriflavine, a clinically aproved drug, inhibits SARS-CoV-2 and other betacoronaviruses. *bioRxiv*, 2021.

[156] Yanni Lv, Saisai Wang, Peida Liang, Yamin Wang, Xin Zhang, Qianqian Jia, Jia Fu, Shengli Han, and Langchong He. Screening and evaluation of anti-SARS-CoV-2 components from ephedra sinica by ace2/cmc-hplc-it-tof-ms approach. *Analytical and Bioanalytical Chemistry*, 413(11):2995–3004, 2021.

[157] Žiko B. Milanović, Marko R. Antonijević, Ana D. Amić, Edina H. Avdović, Dušan S. Dimić, Dejan A. Milenković, and Zoran S. Marković. Inhibitory activity of quercetin, its metabolite, and standard antiviral drugs towards enzymes essential for SARS-CoV-2: the role of acid–base equilibria. *RSC Adv.*, 11:2838–2847, 2021.

[158] Yue-Yu Gu, Min Zhang, Huan Cen, Yi-Fan Wu, Zhaoyu Lu, Fuhua Lu, Xu-Sheng Liu, and Hui-Yao Lan. Quercetin as a potential treatment for COVID-19-induced acute kidney injury: Based on network pharmacology and molecular docking study. *PLOS ONE*, 16(1):1–17, 01 2021.

[159] Shalja Verma and Anand Kumar Pandey. Factual insights of the allosteric inhibition mechanism of SARS-CoV-2 main protease by quercetin: an in silico analysis. *3 Biotech*, 11(2):67, 2021.

[160] Ali Saeedi-Boroujeni and Mohammad-Reza Mahmoudian-Sani. Anti-inflammatory potential of quercetin in COVID-19 treatment. *Journal of Inflammation*, 18(1):3, 2021.

[161] Olga Abian, David Ortega-Alarcon, Ana Jimenez-Alesanco, Laura Ceballos-Laita, Sonia Vega, Hugh T. Reyburn, Bruno Rizzuti, and Adrian Velazquez-Campoy. Structural stability of SARS-CoV-2 3CLpro and identification of quercetin as an inhibitor by experimental screening. *International Journal of Biological Macromolecules*, 164:1693–1703, 2020.

[162] Michael A. Kiebish, Punit Shah, Rangaprasad Sarangarajan, Vivek K. Vishnudas, Stephane Gesta, Poornima K. Tekumalla, Chas Bountra, Elder Granger, Eric Schadt, Leonardo O. Rodrigues, and Niven R. Narain. Bayesian model infers drug repurposing candidates for treatment of COVID-19. *Applied Sciences*, 11(6), 2021.

[163] Son Tung Ngo, Hung Minh Nguyen, Le Thi Thuy Huong, Pham Minh Quan, Vi Khanh Truong, Nguyen Thanh Tung, and Van V. Vu. Assessing potential inhibitors of SARS-CoV-2 main protease from available drugs using free energy perturbation simulations. *RSC Adv.*, 10:40284–40290, 2020.

[164] Bernhard Ellinger, Denisa Bojkova, Andrea Zaliani, Jindrich Cinatl, Carsten Claussen, Sandra Westhaus, Oliver Keminer, Jeanette Reinshagen, Maria Kuzikov, Markus Wolf, Gerd Geisslinger, Philip Gribbon, and Sandra Ciesek. A SARS-CoV-2 cytopathicity dataset generated by high-content screening of a large drug repurposing collection. *Scientific Data*, 8(1):70, 2021.

[165] Ibrahim E. Awad, Abd Al-Aziz A. Abu-Saleh, Sweta Sharma, Arpita Yadav, and Raymond A. Poirier. High-throughput virtual screening of drug databanks for potential inhibitors of SARS-CoV-2 spike glycoprotein. *Journal of Biomolecular Structure and Dynamics*, 0(0):1–14, 2020. PMID: 33103586.

[166] Hussain Mustatab Wahedi, Sajjad Ahmad, and Sumra Wajid Abbasi. Stilbene-based natural compounds as promising drug candidates against COVID-19. *Journal of Biomolecular Structure and Dynamics*, 0(0):1–10, 2020. PMID: 32345140.

[167] Mark A. Marinella. Indomethacin and resveratrol as potential treatment adjuncts for SARS-CoV-2/COVID-19. *International Journal of Clinical Practice*, 74(9):e13535, 2020.

[168] Justine R. Horne and Marie-Claude Vohl. Biological plausibility for interactions between dietary fat, resveratrol, ace2, and sars-cov illness severity. *American Journal of Physiology-Endocrinology and Metabolism*, 318(5):E830–E833, 2020. PMID: 32310688.

[169] B.M. Ellen ter, N. Dinesh Kumar, E.M. Bouma, B. Troost, D.P.I. Pol van de, H.H. Ende van der Metselaar, L. Apperloo, D. Gosliga van, M. Berge van den, M.C. Nawijn, P.H.J. Voort van der, J. Moser, I.A. Rodenhuis-Zybert, and J.M. Smit. Resveratrol and pterostilbene potently inhibit SARS-CoV-2 infection in vitro. *bioRxiv*, 2020.

[170] Minghui Yang, Jinli Wei, Ting Huang, Luping Lei, Chenguang Shen, Jinzhi Lai, Min Yang, Lei Liu, Yang Yang, Guoshi Liu, and Yingxia Liu. Resveratrol inhibits the replication of severe acute respiratory syndrome coronavirus 2 (SARS-CoV-2) in cultured vero cells. *Phytotherapy Research*, 35(3):1127–1129, 2021.

[171] Alessia Catalano, Domenico Iacopetta, Michele Pellegrino, Stefano Aquaro, Carlo Franchini, and Maria Stefania Sinicropi. Diarylureas: Repositioning from antitumor to antimicrobials or multi-target agents against new pandemics. *Antibiotics*, 10(1), 2021.

[172] Valentina Giudice, Pasquale Pagliano, Alessandro Vatrella, Alfonso Masullo, Sergio Poto, Benedetto Maria Polverino, Renato Gammaldi, Angelantonio Maglio, Carmine Sellitto, Carolina Vitale, Bianca Serio, Bianca Cuffa, Anna Borrelli, Carmine Vecchione, Amelia Filippelli, and Carmine Selleri. Combination of ruxolitinib and eculizumab for treatment of severe SARS-CoV-2-related acute respiratory distress syndrome: A controlled study. *Frontiers in Pharmacology*, 11:857, 2020.

[173] F. Heidel and A. Hochhaus. Holding covid in check through jak? the mpn-approved compound ruxolitinib as a potential strategy to treat SARS-CoV-2 induced systemic hyperinflammation. *Leukemia*, 34(7):1723–1725, 2020.

[174] Andreas Neubauer, Thomas Wiesmann, Claus F. Vogelmeier, Elisabeth Mack, Chrysanthi Skevaki, Christine Gaik, Christian Keller, Jens Figiel, Kristina Sohlbach, Caroline Rolfes, Harald Renz, Hinnerk Wulf, and Andreas Burchert. Ruxolitinib for the treatment of SARS-CoV-2 induced acute respiratory distress syndrome (ards). *Leukemia*, 34(8):2276–2278, 2020.

[175] A. D'Alessio, P. Del Poggio, F. Bracchi, G. Cesana, N. Sertori, D. Di Mauro, A. Fargnoli, M. Motta, C. Giussani, P. Moro, G. Vitale, M. Giacomini, and G. Borra. Low-dose ruxolitinib plus steroid in severe SARS-CoV-2 pneumonia. *Leukemia*, 35(2):635–638, 2021.

[176] Bakiye Goker Bagca and Cigir Biray Avci. The potential of JAK/STAT pathway inhibition by ruxolitinib in the treatment of COVID-19. *Cytokine & Growth Factor Reviews*, 54:51–61, 2020. Therapeutic Opportunities in the Management of COVID-19.

[177] F. La Rosée, H. C. Bremer, I. Gehrke, A. Kehr, A. Hochhaus, S. Birndt, M. Fellhauer, M. Henkes, B. Kumle, S. G. Russo, and P. La Rosée. The janus kinase 1/2 inhibitor ruxolitinib in COVID-19 with severe systemic hyperinflammation. *Leukemia*, 34(7):1805–1815, 2020.

[178] Yang Cao, Jia Wei, Liang Zou, Tiebin Jiang, Gaoxiang Wang, Liting Chen, Liang Huang, Fankai Meng, Lifang Huang, Na Wang, Xiaoxi Zhou, Hui Luo, Zekai Mao, Xing Chen, Jungang Xie, Jing Liu, Hui Cheng, Jianping Zhao, Gang Huang, Wei Wang, and Jianfeng Zhou. Ruxolitinib in treatment of severe coronavirus disease 2019 (COVID-19): A multicenter, single-blind, randomized controlled trial. *Journal of Allergy and Clinical Immunology*, 146(1):137–146.e3, July 2020.

[179] Nazanin Fathi and Nima Rezaei. Lymphopenia in COVID-19: Therapeutic opportunities. *Cell Biology International*, 44(9):1792–1797, 2020.

[180] Toluwase Hezekiah Fatoki, Omodele Ibraheem, Ibukun Oladejo Ogunyemi, Afolabi Clement Akinmoladun, Harriet U. Ugboko, Catherine Joke Adeseko, Oladoja A. Awofisayo, Sunday Joseph Olusegun, and Jesupemi Mercy Enibukun. Network analysis, sequence and structure dynamics of key proteins of coronavirus and human host, and molecular docking of selected phytochemicals of nine medicinal plants. *Journal of Biomolecular Structure and Dynamics*, 0(0):1–23, 2020. PMID: 32686993.

[181] Yadi Zhou, Yuan Hou, Jiayu Shen, Yin Huang, William Martin, and Feixiong Cheng. Network-based drug repurposing for novel coronavirus 2019-ncov/SARS-CoV-2. *Cell Discovery*, 6(1):14, 2020.

[182] Loukman Omarjee, Anne Janin, Frédérique Perrot, Bruno Laviolle, Olivier Meilhac, and Guillaume Mahe. Targeting t-cell senescence and cytokine storm with rapamycin to prevent severe progression in COVID-19. *Clinical Immunology*, 216:108464, 2020.

[183] Amjad Husain and Siddappa N. Byrareddy. Rapamycin as a potential repurpose drug candidate for the treatment of COVID-19. *Chemico-Biological Interactions*, 331:109282, 2020.

[184] Paolo Fagone, Rosella Ciurleo, Salvo Danilo Lombardo, Carmelo Iacobello, Concetta Ilenia Palermo, Yehuda Shoenfeld, Klaus Bendtzen, Placido Bramanti, and Ferdinando Nicoletti. Transcriptional landscape of SARS-CoV-2 infection dismantles pathogenic pathways activated by the virus, proposes unique sex-specific differences and predicts tailored therapeutic strategies. *Autoimmunity Reviews*, 19(7):102571, 2020. Special issue COVID19 and Autoimmunity.

[185] Kevin Klann, Denisa Bojkova, Georg Tascher, Sandra Ciesek, Christian Münch, and Jindrich Cinatl. Growth factor receptor signaling inhibition prevents SARS-CoV-2 replication. *Molecular Cell*, 80(1):164–174.e4, 2020.

[186] Min Zhang, Chao Feng, Xingchen Zhang, Shuofeng Hu, Yuan Zhang, Min Min, Bing Liu, Xiaomin Ying, and Yan Liu. Susceptibility factors of stomach for SARS-CoV-2 and treatment implication of mucosal protective agent in COVID-19. *Frontiers in Medicine*, 7:1046, 2021.

[187] B. A. N. K. SARBASHRI, Nipa Basak, G. V. Girish, Subrata Kumar De, and Smarajit Maiti. In-silico analysis of potential interaction of drugs and the SARS-CoV-2 spike protein. *Research Square*, 2021.

[188] Clarisse Salgado-Benvindo, Melissa Thaler, Ali Tas, Natacha S. Ogando, Peter J. Bredenbeek, Dennis K. Ninaber, Ying Wang, Pieter S. Hiemstra, Eric J. Snijder, and Martijn J. van Hemert. Suramin inhibits SARS-CoV-2 infection in cell culture by interfering with early steps of the replication cycle. *Antimicrobial Agents and Chemotherapy*, 64(8), 2020.

[189] Wei Zhu, Miao Xu, Catherine Z. Chen, Hui Guo, Min Shen, Xin Hu, Paul Shinn, Carleen Klumpp-Thomas, Samuel G. Michael, and Wei Zheng. Identification of SARS-CoV-2 3cl protease inhibitors by a quantitative high-throughput screening. *ACS Pharmacol. Transl. Sci.*, 3(5):1008–1016, October 2020.

[190] Raphael J. Eberle, Danilo S. Olivier, Marcos S. Amaral, Ian Gering, Dieter Willbold, Raghuvir K. Arni, and Monika A. Coronado. The repurposed drugs suramin and quinacrine cooperatively inhibit SARS-CoV-2 3clpro in vitro. *Viruses*, 13(5), 2021.

[191] Zharko Daniloski, Tristan X. Jordan, Hans-Hermann Wessels, Daisy A. Hoagland, Silva Kasela, Mateusz Legut, Silas Maniatis, Eleni P. Mimitou, Lu Lu, Evan Geller, Oded Danziger, Brad R. Rosenberg, Hemali Phatnani, Peter Smibert, Tuuli Lappalainen, Benjamin R. tenOever, and Neville E. Sanjana. Identification of required host factors for SARS-CoV-2 infection in human cells. *Cell*, 184(1):92–105.e16, January 2021.

[192] Georgia Ragia and Vangelis G. Manolopoulos. Inhibition of SARS-CoV-2 entry through the ace2/tmprss2 pathway: a promising approach for uncovering early COVID-19 drug therapies. *European journal of clinical pharmacology*, 76(32696234):1623–1630, December 2020.

[193] Xu Li, Jinchao Yu, Zhiming Zhang, Jing Ren, Alex E Peluffo, Wen Zhang, Yujie Zhao, Jiawei Wu, Kaijing Yan, Daniel Cohen, and Wenjia Wang. Network bioinformatics analysis provides insight into drug repurposing for COVID-19. *Medicine in Drug Discovery*, page 100090, 2021.

[194] Zhenming Jin, Xiaoyu Du, Yechun Xu, Yongqiang Deng, Meiqin Liu, Yao Zhao, Bing Zhang, Xiaofeng Li, Leike Zhang, Chao Peng, Yinkai Duan, Jing Yu, Lin Wang, Kailin Yang, Fengjiang Liu, Rendi Jiang, Xinglou Yang, Tian You, Xiaoce Liu, Xiuna Yang, Fang Bai, Hong Liu, Xiang Liu, Luke W. Guddat, Wenqing Xu, Gengfu Xiao, Chengfeng Qin, Zhengli Shi, Hualiang Jiang, Zihe Rao, and Haitao Yang. Structure of mpro from SARS-CoV-2 and discovery of its inhibitors. *Nature*, 582(7811):289–293, 2020.

[195] Chunlong Ma, Yanmei Hu, Julia Alma Townsend, Panagiotis I. Lagarias, Michael Thomas Marty, Antonios Kolocouris, and Jun Wang. Ebselen, disulfiram, carmofur, px-12, tideglusib, and shikonin are nonspecific promiscuous SARS-CoV-2 main protease inhibitors. *ACS Pharmacol. Transl. Sci.*, 3(6):1265–1277, December 2020.

[196] Sven Ullrich and Christoph Nitsche. The SARS-CoV-2 main protease as drug target. *Bioorganic & Medicinal Chemistry Letters*, 30(17):127377, 2020.

[197] Donald C. Hall and Hai-Feng Ji. A search for medications to treat COVID-19 via in silico molecular docking models of the SARS-CoV-2 spike glycoprotein and 3cl protease. *Travel Medicine and Infectious Disease*, 35:101646, 2020.

[198] Lucas Walz, Avi J. Cohen, Andre P. Rebaza, James Vanchieri, Martin D. Slade, Charles S. Dela Cruz, and Lokesh Sharma. Jak-inhibitor and type i interferon ability to produce favorable clinical outcomes in COVID-19 patients: a systematic review and meta-analysis. *BMC Infectious Diseases*, 21(1):47, 2021.

[199] Alan C-Y. Hsu, Guoqiang Wang, Andrew T. Reid, Punnam Chander Veerati, Prabuddha S. Pathinayake, Katie Daly, Jemma R. Mayall, Philip M. Hansbro, Jay C. Horvat, Fang Wang, and Peter A. Wark. SARS-CoV-2 spike protein promotes hyper-inflammatory response that can be ameliorated by spike-antagonistic peptide and fda-approved er stress and map kinase inhibitors in vitro. *bioRxiv*, 2020.

[200] Motonori Tsuji. Potential anti-SARS-CoV-2 drug candidates identified through virtual screening of the chembl database for compounds that target the main coronavirus protease. *FEBS Open Bio*, 10(6):995–1004, 2020.

[201] Yiming Zhang, Rory A Greer, Yuwei Song, Hrithik Praveen, and Yuhua Song. In silico identification of available drugs targeting cell surface bip to disrupt SARS-CoV-2 binding and replication: Drug repurposing approach. *European Journal of Pharmaceutical Sciences*, 160:105771, 2021.

[202] YW Chen, CPB Yiu, and KY Wong. Prediction of the SARS-CoV-2 (2019-ncov) 3c-like protease (3clpro) structure: virtual screening reveals velpatasvir, ledipasvir, and other drug repurposing candidates [version 2; peer review: 3 approved]. *F1000Research*, 9(129), 2020.

[203] Thai-Hoang Pham, Yue Qiu, Jucheng Zeng, Lei Xie, and Ping Zhang. A deep learning framework for high-throughput mechanism-driven phenotype compound screening and its application to COVID-19 drug repurposing. *Nature Machine Intelligence*, 3(3):247–257, 2021.

[204] Valentina L. Kouznetsova, Aidan Zhang, Mahidhar Tatineni, Mark A. Miller, and Igor F. Tsigelny. Potential COVID-19 papain-like protease pl(pro) inhibitors: repurposing fda-approved drugs. *PeerJ*, 8(32999768):e9965–e9965, September 2020.

[205] Giulia Fiscon, Federica Conte, Lorenzo Farina, and Paola Paci. Saverunner: A network-based algorithm for drug repurposing and its application to COVID-19. *PLOS Computational Biology*, 17(2):1–30, 02 2021.

[206] VL Kouznetsova, C Kellogg, A Zhang, et al. Pharmacophore investigations of potential covid-19 rna polymerase (rdrp) inhibitors: Repurposing fda-approved drugs. *Microbiology & Infectious Diseases*, 5(1):1–9, 2021.

[207] Nina Verstraete, Giuseppe Jurman, Giulia Bertagnolli, Arsham Ghavasieh, Vera Pancaldi, and Manlio De Domenico. Covmulnet19, integrating proteins, diseases, drugs, and symptoms: A network medicine approach to COVID-19. *Network and Systems Medicine*, 3(1):130–141, 2020.

[208] Santiago Thibaud, Douglas Tremblay, Sheena Bhalla, Brittney Zimmerman, Keith Sigel, and Janice Gabrilove. Protective role of bruton tyrosine kinase inhibitors in patients with chronic lymphocytic leukaemia and COVID-19. *British Journal of Haematology*, 190(2):e73–e76, 2020.
