# Supplementary material for: Network analytics for drug repurposing in COVID-19
Source: Brief Bioinform. 2021 Dec 7;23(1):bbab490. doi: 10.1093/bib/bbab490 (PMC8690228; doi:10.1093/bib/bbab490)
Supplement: supplementary_information_bbab490 [file supplementary_information_bbab490.pdf]

1 The COVID-19 directed protein-protein interaction networks

We constructed two directed protein-protein interaction (PPI) networks, one for each of the datasets of [1] with the essential host factors required for the SARS-CoV-2 infection at MOI 0.01 and MOI 0.3, resp. We narrowed the interaction networks to include only the proteins upstream of the essential proteins of [1] and downstream of the drug-targets from DrugBank [2], at a distance of at most two interactions from either of these sets. We added between these proteins all interactions we found in KEGG, OmniPath and SIGNOR. Any essential protein or drug target that remained isolated in the network was removed. This way, we narrowed the search for possible influences from drug-targets to essential proteins to those acting along very short distances in the networks. The results of the topological analyses of the networks are illustrated in Table 1 and the results of the centrality analyses are in Table 2.

We then calculated the intersection of the two sets of protein identifiers, obtaining all proteins that are on a signaling path from a drug target to an essential gene, at a downstream distance from the drug targets at most 2 and at an upstream distance from the essential genes at most 2. We obtained the networks by considering all the directed interactions between these proteins, the drug target proteins, and the essential proteins. The same method was applied to both MOI datasets, the difference being in the sets of host factors. This obviously also led to differences in the intermediate proteins from the drug targets to the host factors.

Table 1: Statistics of the two protein-protein directed interaction networks. **N**: number of nodes; **E**: number of edges; **T**: number of host factors in the network; **DT**: number of drug-targets; **AD**: network average degree; **D**: network diameter; **CC**: number of connected components.

MOI	N	E	T	DT	AD	D	CC
0.01	2491	23746	70	1010	17.305	10	2
0.3	2532	24105	62	1008	17.296	10	1

We analyzed the topology of our networks using several centrality measures: degree (total degree, in-degree, and out-degree) centrality, closeness-, betweenness-, and harmonic-centrality [3]. We identified the top 3 ranked

Table 2: Top ranked proteins in our networks, based on their centrality measures

**Total degree**	**In-degree**	**Out-degree**	**Closeness**	**Betweenness**	**Harmonic**
SRC	TP53	PRKCA	TP53	SRC	TP53
GSK3B	PTK2	SRC	CTNNB1	TP53	CTNNB1
PRKCA	CTNNB1	GSK3B/CDK1	SRC	AKT1	SRC

proteins in each network, based on each of these centrality measures, with the results collected in Table 2. The top-ranked proteins were virtually identical for both networks, with only one difference in the proteins with the highest out-degree: GSK3B was ranked third in the MOI 0.01 network, while CDK1 was similarly ranked in the MOI 0.3 network.

The proteins with the highest total- and out- degree were PRKCA, SRC and GSK3B, out of which SRC and GSK3B have been shown to be potential targets for COVID-19 [4].

Another protein of interest is TP53, which was top 2-ranked in all the other centrality measures. TP53 is known to be active in down-regulating the SARS-CoV replication [5]. Other highly ranked central proteins include AKT1, shown to be of significance to SARS-CoV-2 [6], CDK1, identified as potential therapeutic option in for COVID-19 [7], and CTNNB1, linked to co-morbidities associated with COVID-19 severity [8].

2 Target network controllability

Network controllability is about being able to induce arbitrary changes to the variables of a network through suitable changes applied to a (small) set of input nodes. It is a method applicable to linear networks. Such a network with $n \geq 2$ nodes can be represented as an n-node directed (weighted) graph, and also as a matrix $A \in \mathbb{R}^{n \times n}$ describing the weights of each possible interaction between the nodes. A directed network is said to be controllable from a set of input nodes if for any desired initial and final numerical configurations of the nodes in the network, there exists a suitable set of input functions applicable to the input nodes that induce the desired change of configuration [9]. The methodology accommodates also the possibility of imposing such a desired change of configuration to a target subset of nodes in the network [10]. The target network controllability problem asks to find a minimum set of input nodes controlling the target subset.

Formally, we have a dynamical system defined by the system of differential equations $dx(t)/dt = Ax(t)$, where $x : \mathbb{R} \to \mathbb{R}^n$ and $x(t)$ represents

the configuration of the n nodes at time t. Let V define the n-set of nodes of the network. The set of input functions acting on some size-m subset $I \subseteq V$ of the network can be seen as an m-dimensional input vector u of real functions, $u : \mathbb{R} \to \mathbb{R}^m$. Thus, the dynamical system under the influence of these functions is formally described by the system:

$$dx(t)/dt = Ax(t) + B_I u(t), \tag{1}$$

where $B_I \in \mathbb{R}^{n \times m}$ is the characteristic matrix associated to set I.

Within this theoretical setting, the target controllability problem for a dynamical system defined by matrix $A \in \mathbb{R}^{n \times n}$ and target set $T \subseteq V$, $|T| = l$, is defined as follows: find the smallest $m \leq n$ and input set (a.k.a controller) $I \subseteq V$, $|I| = m$, such that for any $x(0) \in \mathbb{R}^n$ and any $\alpha \in \mathbb{R}^l$, there is an input vector $u : \mathbb{R} \to \mathbb{R}^m$ such that the solution $\tilde{x}$ of 1 eventually coincides with α on its T-components, i.e., $C_T \tilde{x}(\tau) = \alpha$, for some $\tau \geq 0$, where C_T is the characteristic matrix of the target set T. Although the problem is known to be computationally hard [11], some efficient approximation algorithms were provided in [11, 12].

Motivated by bio-medical applications of network controllability, a new layer of optimization has been added to the above formulation. Namely, the input-constrained target controllability problem [13, 14] asks to minimize the size of the controller for a given target T within a network, by maximizing in the same time the use of so-called preferred input nodes within the controller, i.e., elements of a previously defined subset $P \subseteq V$ of preferred input nodes. Indeed such a subset P can be associated in the bio-medical setting to those proteins/genes known to be the targets of available drugs. Thus, it is useful not only to minimize the controller of a targeting subset of focused nodes, but in doing so to rely as much as possible to the use of already available drugs. It is this particular setting of the controllability problem that has been used as the underlying methodological approach in this research.

We used the NetControl4BioMed web application [15] and its Greedy algorithm described in [14] to apply the target controllability analysis on our networks. The analysis setup is the following: we use the set of host factors in each network as controllability targets and we aim to identify a minimal set of drug-targetable proteins that control the host factors. As the set of control targets we used the set of host factors in each of the two MOI networks and as the set of prefered input nodes we chose the set of drug targetable proteins. We limited the analysis to using control paths of maximum length 3 (i.e. there are at most three interactions between a drug-target and a host factor that it controls), to minimize the potential

dissipation of a drug's effects along the pathway. The analyses are stochastic, which means that the same input can lead to different control solutions.

For each control analysis, we defined the control score of a drug-target as the number of host factors it controls in that analysis. We then defined the efficiency score of a drug as the maximum control score of any of its drug targets. Thus, for each analysis, we identified the set of drugs with the maximum efficiency score. The actual value of the maximum score may vary between analyses. We then defined the efficiency score of a drug as the maximum control score of its set of drug targets within any of the stochastic analyses we ran. Thus, in each analysis, we identified the drugs with the maximum efficiency score. The actual value of the maximum score may vary between analyses.

We ran repeatedly the network controllability analysis for each network until no new top ranked drug is found within three consecutive runs.

3 Repurposable drugs: other compounds

There are several natural compounds in our list such as resveratrol, caffeine, genistein, ellagic acid, alvocidib, quercetin, emodin. Of these, alvocidib, quercetin, resveratrol and genistein contains flavonoids. There are some antiallergic, anti-inflammatory, antiviral, antioxidant activities reported for some flavonoids [16], but also antithrombogenic effects because they can increase fibrinolysis [17].

We also obtained lithium-based mood stabilizers as GSK3B inhibitors (lithium carbonate and lithium citrate). Targeting GSK3B is also tideglusib, a drug investigated for use in Alzheimer's disease.

Additionally, we also obtained some investigational compounds that act on MAPK14: KC706, talmapimod, PH-797804, VX-702. Inhibition of p38/MAPK signaling is beneficial in SARS, DENV and IAV, and in the case of SARS-CoV-2 it suppresses cytokine production and affects viral replication [18]. PH-797804 was also discussed in [19] for potential use to limit lung damage.

Isoprenaline is a heart stimulant, used especially in emergencies, in heart shock, but can also be used by inhalation in asthma and chronic bronchitis. In our results it came up through its inhibitory action on MAPK1.

Our analysis identified some compounds that should not be recommended for use in practice. Antibiotics are usually not recommended in a viral infection. We obtained minocycline, a drug from the class of tetracycline that is known to be preferred to macrolides because its representatives are less toxic. Another compound that is difficult to use is cefazolin, a cephalosporin

that acts on the Il2 target. Invalid compounds are also those that act as a topical cream (for example, ingenol mebutate) or as eye drops (for example, netarsudil). Sucralfate is another false positive because it does not act systemically, but could help to prevent further transmission from the stomach to the intestine.

4 Clinical trials

Ibuprofen was obtained in our analyses due to its effect on BCL2. It is currently included in two clinical trials (NCT04382768, NCT04334629) to be evaluated for its ability to reduce the severity and progression of lung injury.

The followings drugs were obtained only on the MOI 0.01 network: argatroban, baricitinib, bivalirudin, estradiol, estradiol cypionate, imatinib, lidocaine, lithium carbonate, nafamostat, ruxolitinib, suramin, tamoxifen, tofacitinib, zanubrutinib. Argatroban and bivalirudin may help in COVID-19 due to their action on coagulation, especially when heparin resistance is involved [20, 21]. Argatroban is included in NCT04406389 clinical trail together with enoxaparin, fondapariniux and unfractioned heparin to establish if they can reduce mortality, while bivalirudin is included in NCT04445935, to see if it could prevent clotting better than low molecular weight heparin and support the dissolution of existing clots. Nafamostat, another F2 inhibitor, could be used due to its antiviral, anti-inflammatory and anti-coagulation properties (NCT04418128). According to clinical studies, estradiol may control the inflammmation (IRCT20150716023235N15), while lidocaine may impact the gas exchange and inflammation in acute respiratory distress syndrome (NCT04609865). Tofacitinib, a selective immunosuppressant used in rheumatoid arthritis that is also being investigated for its use in preventing transplant rejection [2], is included in clinical studies to find out if it is suitable to moderate or severe cases (NCT04469114, NCT04415151). Imatinib may have some antiviral properties due to lysosomal alkalization (NCT04394416), while zanubrutinib may lower elevated levels of pro-inflammatory cytokines [22, 23], and it is investigated as it could increase the respiratory failure-free survival rate (NCT04382586). Lithium carbonate could also have an effect on pro-inflammatory cytokines [24], and it is included in several clinical trials, such as: CTRI/2020/06/026193, IRCT20081019001369N5, IRCT20130812014333N147. Tamoxifen is administred in two clinical studies, but only in combination with other drugs (NCT04389580, NCT04568096).

The drugs obtained on both networks are: acetylsalicylic acid, arsenic trioxide, dasatinib, genistein, minocycline, quercetin, resveratrol and sirolimus. Aspirin has been investigated in COVID-19 clinical trials for its use in coagulation problems (NCT04363840, NCT04498273), but may inhibit virus replication and reduce lung injury (NCT04365309). It inhibits the platelet aggregation due to its action on COX-1. However, we did not identify it through its effect on COX-1, but through its action on TP53, on RPS6KA3, on PRKAA1, and on MAPK1. The only drug target obtained for both MOI network analyses is TP53. The action on TP53 could be related to the aspirin-induced inhibition of adipogenesis [25]; obesity can aggravate COVID-19.

Other clinical trials have mentioned possible effects for the followings drugs: genistein (could be efficient in pulmonary fibrosis in patients discharged from hospital (NCT04482595)), quercetin (may help because it has antiviral activities in SARS infection and antioxidant properties (NCT04377789)), resveratrol (could have anti-fibrotic effects (NCT04799743)), sirolimus (may help in reducing lung injury/acute respiratory distress syndrome (NCT04482712)), dasatinib (may help reduce the strong inflammation (NCT04830735)).

5 Supplementary figures

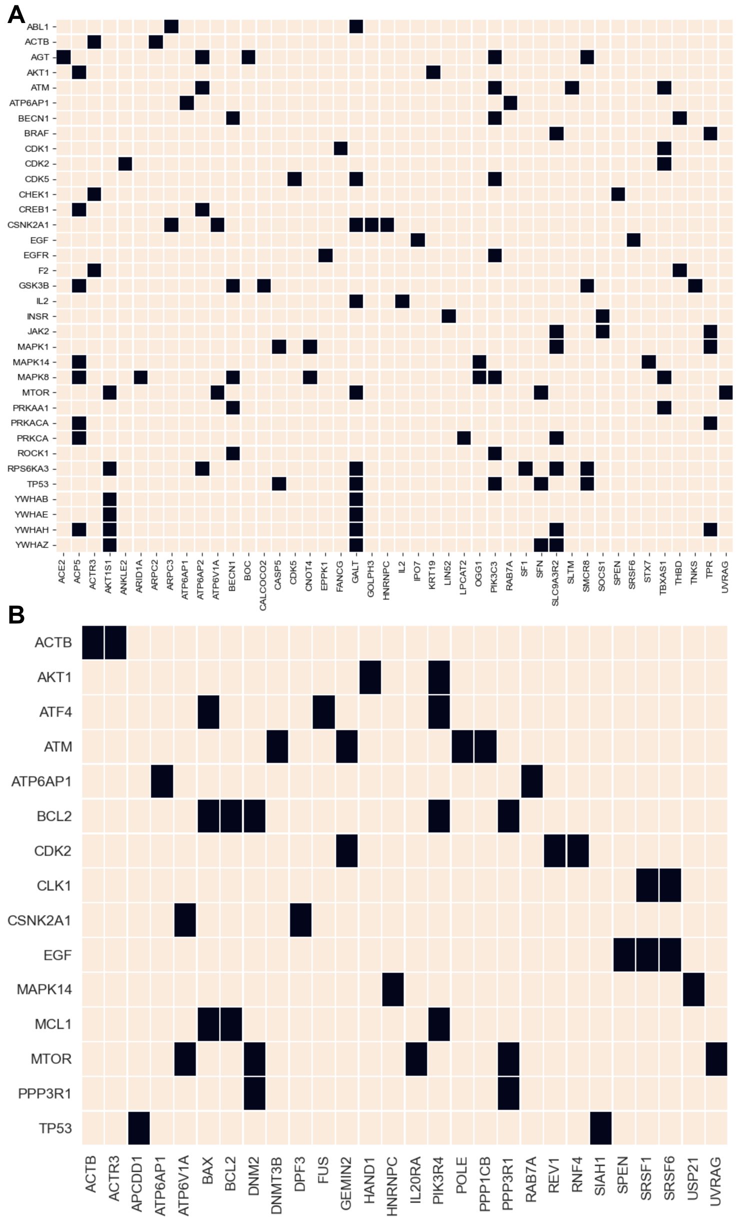

Figure 1: Correspondence between drug-target and SARS-CoV-2 host factors. A: MOI 0.01, **B**: MOI 0.3. **On the horizontal axis**: the top 200 SARS-CoV-2 host factors ([1]) included in the interaction network, **On the vertical axis**: the drug targets included in the interaction network. A black rectangle marks a drug target identified by our analysis to control the corresponding host factor.

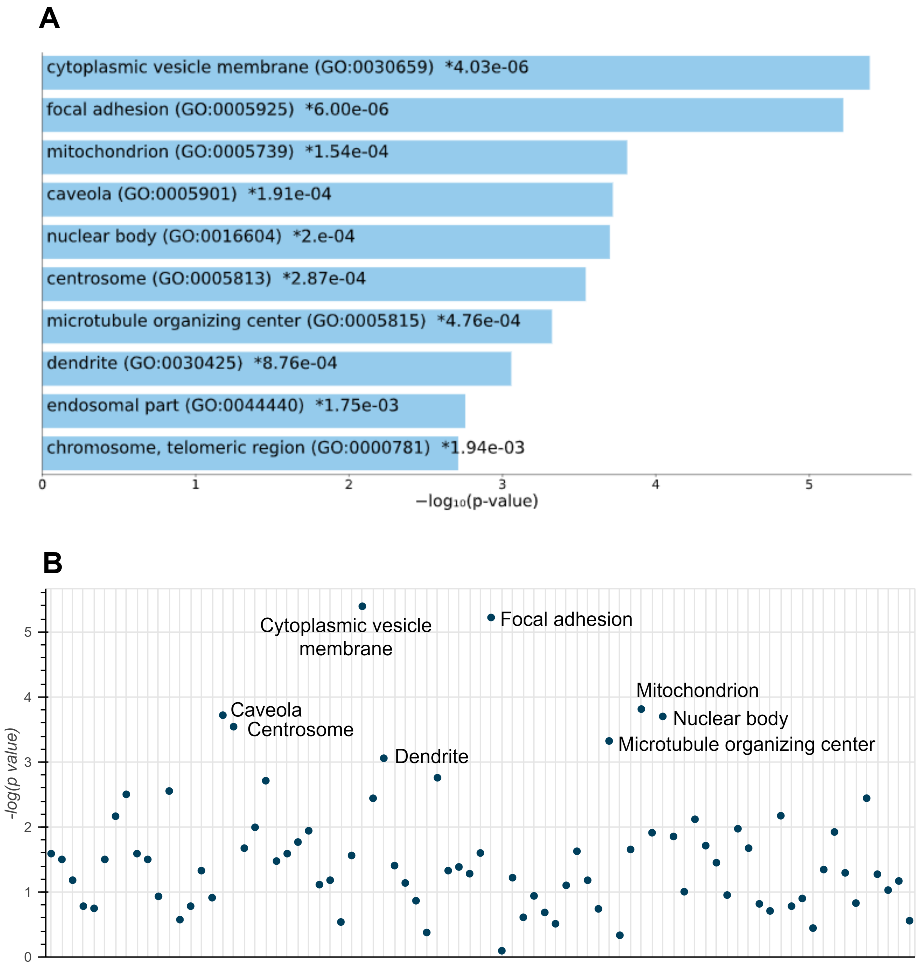

Figure 2: Localization at the cell level [26, 27]. **A**: GO Cellular Component 2018, **B**: Manhattan plot.

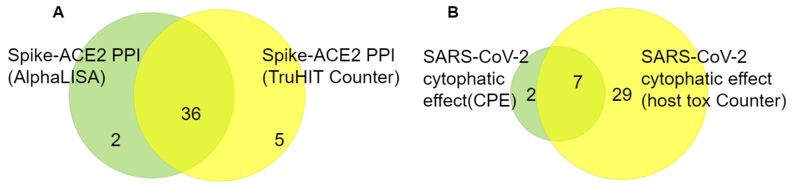

Figure 3: The overlap between drugs active in the assay and in the counter-assay. **A**: spike-ACE2 PPI in assay and counter-assay, **B**: cytophatic effect in assay and counter-assay. **Color code**: green – drugs active in the assay, yellow – drugs active in the counter-assay.

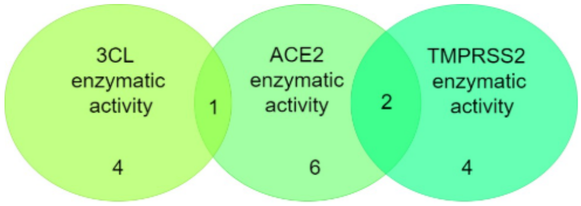

Figure 4: The effect on enzymatic activity. **A**: Euler diagram on enzymatic activity, **B**: 3CLpro enzymatic activity, **C**: TMPRSS2 enzymatic activity, **D**: ACE2 enzymatic activity.

References

[1] Daniloski Z, Jordan TX, Wessels H-H, et al. Identification of required host factors for SARS-CoV-2 infection in human cells. *Cell*, 184(1):92–105.e16, January 2021.

[2] Wishart DS, Feunang YD, Guo AC, et al. Drugbank 5.0: a major update to the drugbank database for 2018. *Nucleic Acids Research*, 46:1074–1082, 2017.

[3] Liu X, Hong Z, Liu J, et al. Computational methods for identifying the critical nodes in biological networks. *Briefings in Bioinformatics*, 21(2):486–497, 02 2019.

[4] Weisberg E, Parent A, Yang PL, et al. Repurposing of Kinase Inhibitors for Treatment of COVID-19. *Pharm Res*, 37(9):167, Aug 2020.

[5] Ma-Lauer Y, Carbajo-Lozoya J, Hein MY, et al. p53 down-regulates SARS coronavirus replication and is targeted by the SARS-unique domain and PLpro via E3 ubiquitin ligase RCHY1. *Proceedings of the National Academy of Sciences*, 113(35):E5192–E5201, 2016.

[6] Appelberg S, Gupta S, Svensson Akusjärvi S, et al. Dysregulation in Akt/mTOR/HIF-1 signaling identified by proteo-transcriptomics of SARS-CoV-2 infected cells. *Emerging microbes & infections*, 9(1):1748–1760, Dec 2020.

[7] Gargouri M, Alzwi A, Abobaker A. Cyclin dependent kinase inhibitors as a new potential therapeutic option in management of COVID-19. *Medical hypotheses*, 146:110380–110380, Jan 2021.

[8] Dolan ME, Hill DP, Mukherjee G, et al. Investigation of COVID-19 comorbidities reveals genes and pathways coincident with the SARS-CoV-2 viral disease. *Scientific Reports*, 10(1):20848, Nov 2020.

[9] Liu Y-Y, Slotine J-J, and Barabási, A-L. Controllability of complex networks. *Nature*, 2011.

[10] Gao J, Liu Y-Y, D'Souza RM, et al. Target control of complex networks. *Nature Communications*, 5(1):5415, 2014.

[11] Czeizler E, Wu KC, Gratie C, et al . Structural target controllability of linear networks. *IEEE/ACM Transactions on Computational Biology and Bioinformatics*, 15:1217–1228, 2018.

[12] Kanhaiya K, Rogojin V, Kazemi K, et al. NetControl4BioMed: a pipeline for biomedical data acquisition and analysis of network controllability. *BMC Bioinformatics*, 19(7):185, Jul 2018.

[13] Guo W-F, Zhang S-W, Wei Z-G, et al. Constrained target controllability of complex networks. *Journal of Statistical Mechanics: Theory and Experiment*, 2017(6):063402, 2017.

[14] Kanhaiya K, Czeizler E, Gratie C, et al. Controlling directed protein interaction networks in cancer. *Scientific Reports*, 7, 2017.

[15] Popescu V-B, Sanchez-Martin JA, Schacherer D, Safadoust S, Majidi N, Andronescu A, Nedea A, Ion D, Mititelu E, Czeizler E, Petre I. NetControl4BioMed: a web-based platform for controllability analysis of protein–protein interaction networks. *Bioinformatics*, 2021.

[16] Middleton EJ, Kandaswami C. Effects of flavonoids on immune and inflammatory cell functions. *Biochemical pharmacology*, 43:1167–79, Mar 1992.

[17] Nijveldt RJ, van Nood E, van Hoorn DE, et al. Flavonoids: a review of probable mechanisms of action and potential applications. *The American Journal of Clinical Nutrition*, 74(4):418–425, 10 2001.

[18] Bouhaddou M, Memon D, Meyer B, et al. The global phosphorylation landscape of SARS-CoV-2 infection. *Cell*, 182(3):685–712.e19, August 2020.

[19] Gilroy DW, De Maeyer RPH, Tepper M, et al. Treating exuberant, non-resolving inflammation in the lung; implications for acute respiratory distress syndrome and COVID-19. *Pharmacology & Therapeutics*, page 107745, 2020.

[20] Arachchillage DJ, Remmington C, Rosenberg A, et al. Anticoagulation with argatroban in patients with acute antithrombin deficiency in severe COVID-19. *British Journal of Haematology* 190(5): e286–e288, 2020.

[21] Seelhammer TG, Plack D, Lal A, et al. COVID-19 and ECMO: An unhappy marriage of endothelial dysfunction and hemostatic derangements. *Journal of Cardiothoracic and Vascular Anesthesia* 34(12): 3193–3196, 2020.

[22] Thibaud S, Tremblay D, Bhalla S, et al. Protective role of Bruton tyrosine kinase inhibitors in patients with chronic lymphocytic leukaemia and COVID-19. *British Journal of Haematology* 190(2): e73–e76, 2020.

[23] Nascimento Junior JAC, Santos AM, Quintans-Júnior LJ, et al. SARS, MERS and SARS-CoV-2 (COVID-19) treatment: a patent review. *Expert Opinion on Therapeutic Patents* 30(8): 567–579, 2020.

[24] Qaswal AB, Suleiman A, Guzu H, et al. The potential role of lithium as an antiviral agent against SARS-CoV-2 via membrane depolarization: Review and hypothesis. *Scientia Pharmaceutica* 89(1), 2021.

[25] Su YF, Yang SH, Lee YH, et al. Aspirin-induced inhibition of adipogenesis was p53-dependent and associated with inactivation of pentose phosphate pathway. *European Journal of Pharmacology* 738: 101–110, 2014.

[26] Clarke DJB, Jeon M, Stein DJ, et al. Appyters: Turning jupyter notebooks into data-driven web apps. *Patterns*, 2(3):100213, 2021.

[27] Kuleshov MV, Jones MR, Rouillard AD, et al. Enrichr: a comprehensive gene set enrichment analysis web server 2016 update. *Nucleic acids research*, 44(W1):W90–W97, 2016.
